# Regulating the coordination structure of single-atom Fe-N_*x*_C_*y*_ catalytic sites for benzene oxidation

**DOI:** 10.1038/s41467-019-12362-8

**Published:** 2019-09-19

**Authors:** Yuan Pan, Yinjuan Chen, Konglin Wu, Zheng Chen, Shoujie Liu, Xing Cao, Weng-Chon Cheong, Tao Meng, Jun Luo, Lirong Zheng, Chenguang Liu, Dingsheng Wang, Qing Peng, Jun Li, Chen Chen

**Affiliations:** 1 Department of Chemistry, Tsinghua University, Beijing, 100084 China; 2 State Key Laboratory of Heavy Oil Processing, China University of Petroleum (East China), Qingdao, 266580 China; 3 College of Chemistry and Materials Science, Anhui Normal University, Wuhu, 241000 China; 4 Department of Chemistry, Beijing Institute of Technology, Beijing, 100081 China; 5 Center for Electron Microscopy, Tianjin University of Technology, Tianjin, 300384 China; 6 Beijing Synchrotron Radiation Facility, Institute of High Energy Physics, Chinese Academy of Sciences, Beijing, 100049 China

**Keywords:** Catalytic mechanisms, Heterogeneous catalysis, Synthesis and processing

## Abstract

Atomically dispersed metal-N-C structures are efficient active sites for catalyzing benzene oxidation reaction (BOR). However, the roles of N and C atoms are still unclear. We report a polymerization-regulated pyrolysis strategy for synthesizing single-atom Fe-based catalysts, and present a systematic study on the coordination effect of Fe-N_*x*_C_*y*_ catalytic sites in BOR. The special coordination environment of single-atom Fe sites brings a surprising discovery: Fe atoms anchored by four-coordinating N atoms exhibit the highest BOR performance with benzene conversion of 78.4% and phenol selectivity of 100%. Upon replacing coordinated N atoms by one or two C atoms, the BOR activities decrease gradually. Theoretical calculations demonstrate the coordination pattern influences not only the structure and electronic features, but also the catalytic reaction pathway and the formation of key oxidative species. The increase of Fe-N coordination number facilitates the generation and activation of the crucial intermediate O=Fe=O species, thereby enhancing the BOR activity.

## Abstract (web summary)

Atomically dispersed metal-N-C are efficient active site for benzene oxidation but the roles of N and C atoms are still unclear. Here the authors report a highly-active single-atom Fe-based benzene oxidation catalyst and provide deep insights into the structure-activity relationship at atomic level.

## Introduction

Phenol is an important organic chemical intermediate and raw chemical material for numerous industrial products^1,2^, particularly for phenolic resin, cyclohexanol, bisphenol A, epoxy, carbonate, polysulfone, o-cresol, and aniline. Currently, the industrialized process for phenol production from benzene based on multistep and indirect syntheses has several disadvantages^3^, such as complicated synthetic routes, high consumption of raw materials, and serious environmental pollution. The direct oxidation of benzene into phenol by H_2_O_2_ is one of the challenging subjects in the field of green chemistry^4–6^, which is of great industrial and social significance. Therefore, the development of novel and efficient catalysts for the benzene oxidation reaction (BOR) has become a hot topic in green chemistry. A series of catalysts based on non-precious metals have been developed for BOR, but their catalytic performances remain yet to be improved^7,8^. In addition, the compositions of the catalysts are generally complex, and the catalytic efficiencies are relatively low; moreover, the understanding on the catalytic active sites is still scarce^9^, which are not conducive to the study of structure-activity relationship.

In recent years, the concept of single-atom catalysis has become a hot topic^10–16^, with single-atom catalysts (SACs) showing a rapid upsurge in various catalytic fields by virtue of their characteristic high activity and selectivity^17–21^. Moreover, the SACs can also be used as ideal models for exploring the structure-activity relationship at the atomic level. Atomically dispersed metal-N-C materials, usually synthesized by pyrolysis at different temperatures^22^, have been regarded as efficient catalysts for direct catalytic oxidation of benzene to phenol^23^. However, the change of pyrolysis temperature, usually result in some sites with complex structures, such as metal-N_*x* (*x* = 1–4)_, metal-C_*y* (*y* = 1–4)_, and defects in the carbon matrix, which make it very difficult to identify the real catalytic active sites and reaction mechanisms. Although metal-N and metal-C bonds co-existing in the metal-N-C catalysts have been recently reported^24–26^, the roles of N and C atoms in the active sites of metal-N_*x*_C_*y*_ catalysts are still unknown. Therefore, a systematic investigation of the coordination effect of metal-N_*x*_C_*y*_ catalyst is of great significance for understanding the mechanism of catalytic reaction at the atomic level and for guiding the design of more efficient catalysts.

**Fig. 1:**
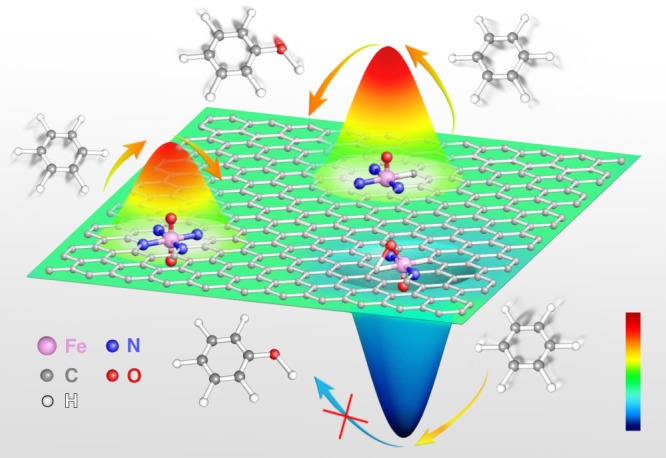
Schematic illustration. The coordination effect of single-atom Fe-N_*x*_C_*y*_ catalytic sites for benzene oxidation

Herein, we report a polymerization-regulated-pyrolysis (PRP) strategy to fabricate a series of single-atom Fe-based catalysts with different Fe coordination environments, so as to explore the coordination-sensitive reactions based on these atomically dispersed catalysts (Fig. 1). By combining a series of experimental studies and density functional theory (DFT) calculations, we found that the single-atom Fe sites anchored by four-coordinating nitrogen atoms exhibit the highest BOR performance with a conversion of 78.4% and a phenol selectivity of 100% at 30 °C, surpassing all the reported BOR catalysts. More interestingly, the BOR activities of single-atom Fe sites catalysts decrease gradually after the coordinating N atoms are replaced by one or two C atoms, revealing a prominent coordination sensitivity. DFT calculations further reveal the intrinsic electronic features accounting for the coordination effect. Specifically, the coordination pattern influences not only the structure and electronic properties of the catalysts, but also the catalytic reaction pathway and the formation of intermediates. The increase of Fe-N coordination number facilitates the generation and activation of the crucial intermediate O=Fe=O species, enhancing the BOR activity. Our findings here not only present a highly-active BOR catalyst, but also provide deep insights for exploring structural-sensitive reaction from the atomic perspective.

## Results

### Synthesis and characterization of Fe-N_*x*_C_*y*_/N-C catalysts

**Fig. 2:**
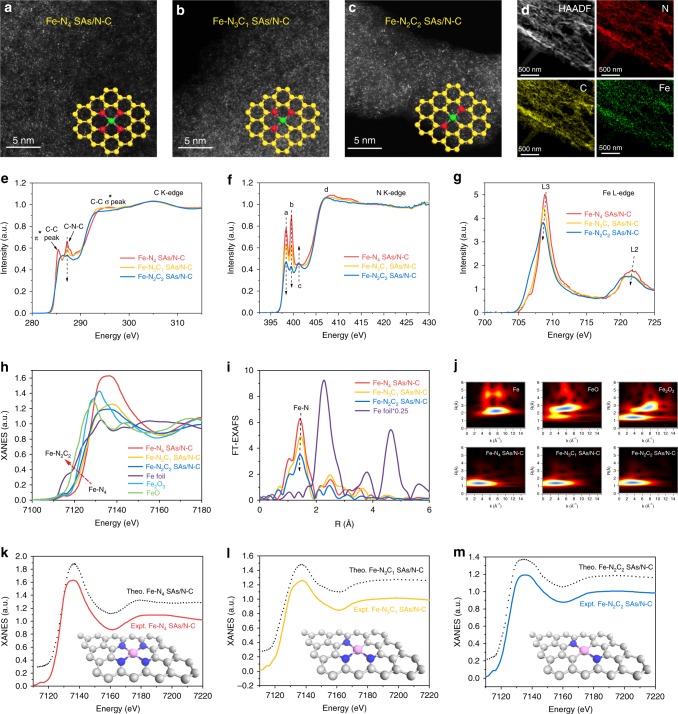
Structure characterization of Fe-N_*x*_C_*y*_ SAs/N-C. **a**–**c** AC-HAADF-STEM images of the as-synthesized **a** Fe-N_4_ SAs/N-C, **b** Fe-N_3_C_1_ SAs/N-C, **c** Fe-N_2_C_2_ SAs/N-C. The insets are the corresponding structure model. The yellow, red and green balls refer to C, N, and Fe atoms, respectively. Scale bar, 5 nm. **d** HAADF-STEM-EDS mapping of the Fe-N_4_ SAs/N-C. Scale bar, 500 nm. **e**–**g** XANES spectra at the **e** C K-edge, **f** N K-edge, and **g** Fe L-edge. **h** XANES spectra at the Fe K-edge of the Fe-N_4_ SAs/N-C, Fe-N_3_C_1_ SAs/N-C, Fe-N_2_C_2_ SAs/N-C, Fe foil, Fe_2_O_3_, FeO. **i** FT k^3^-weighted χ(k)-function of the EXAFS spectra of the Fe-N_4_ SAs/N-C, Fe-N_3_C_1_ SAs/N-C, Fe-N_2_C_2_ SAs/N-C, and Fe foil. **j** WT of the Fe K-edge. **k**–**m** Comparison between the experimental K-edge XANES spectra and the theoretical spectra of **k** Fe-N_4_ SAs/N-C, **l** Fe-N_3_C_1_ SAs/N-C, **m** Fe-N_2_C_2_ SAs/N-C. The grayish-white, blue and pink balls refer to C, N, and Fe atoms, respectively

First, an iron-polyphthalocyanine (FePPc) conjugated polymer network (Supplementary Fig. 1) was synthesized by a low-temperature solvent-free solid-phase method in muffle furnace; then the polymer network was subjected to pyrolysis at specifically regulated temperatures to afford the Fe-N_*x*_C_*y*_ catalysts with different Fe-N coordination numbers (500 °C for Fe-N_4_ SAs/N-C, 600 °C for Fe-N_3_C_1_ SAs/N-C, and 700 °C for Fe-N_2_C_2_ SAs/N-C, in which N-C stands for the N-doped carbon matrix). Further increasing the pyrolysis temperature to 800 °C led to the formation of Fe_3_C (Supplementary Fig. 2); after the Fe_3_C sample was etched with sulphuric acid, Fe nanoparticles (protected by carbon layers) were obtained (Supplementary Fig. 3). For the three Fe-N_*x*_C_*y*_ SAs/N-C catalysts, only a broad peak at 2θ = 26° ((002) plane of carbon) could be observed from their X-ray diffraction (XRD) patterns (Supplementary Fig. 4). Transmission electron microscopy (TEM) images (Supplementary Fig. 5) show that the as-prepared Fe-N_*x*_C_*y*_ SAs/N-C catalysts inherit the layered structure of the FePPc precursor, with no metal NPs observed. The selected area electron diffraction (SAED) images of the as-prepared Fe-N_*x*_C_*y*_ SAs/N-C catalysts (Supplementary Fig. 6) present a ring-like pattern, indicative of a poor crystallinity. As revealed by the high-angle annular dark-field scanning transmission electron microscope (HAADF-STEM) and energy-dispersive X-ray spectroscopy (EDS) mapping images (Fig. 2d, Supplementary Fig. 7), the Fe, C, and N elements in the three products are distributed uniformly over the entire sample. Additionally, the single-atom feature can be directly observed from aberration-corrected HAADF-STEM (AC HAADF-STEM) images, as reflected by the highly dispersed bright dots (Fig. 2a–c). Because the Fe-N and Fe-C bonds have different bond lengths, and Fe-N is a little longer than Fe-C, this provides an opportunity to distinguish between the two coordination modes. Therefore, in order to further distinguish the Fe-N and Fe-C coordination, we compared the distances between two neighboring Fe atoms. As shown in the intensity profiles (Supplementary Fig. 8), the average distances between two neighboring Fe atoms are 4.5 Å for Fe-N_4_ SAs/N-C and 3.5 Å for Fe-N_2_C_2_ SAs/N-C, respectively, which is well consistent with the theoretical results, demonstrating the existence of Fe-C coordination in Fe-N_2_C_2_ SAs/N-C catalyst. The contents of Fe are 3.18, 3.46, 3.06 wt % for Fe-N_4_ SAs/N-C, Fe-N_3_C_1_ SAs/N-C, and Fe-N_2_C_2_ SAs/N-C catalysts, respectively, as confirmed by inductively coupled plasma optical emission spectrometry (ICP-OES). The contents of C, H, N were confirmed by element analysis, as shown in Supplementary Table 1. It can be seen that the total N contents decrease while the total C contents increase gradually from Fe-N_4_ SAs/N-C to Fe-N_2_C_2_ SAs/N-C. To assess the thermal stability of the Fe-N_4_ SAs/N-C, Fe-N_3_C_1_ SAs/N-C, and Fe-N_2_C_2_ SAs/N-C catalysts, we carried out the thermogravimetry coupled with mass spectrometry (TG-MS) analysis. Supplementary Fig. 9 show the TG-DSC curves and the corresponding MS results (*m/z* = 18, 44 are assigned to H_2_O and CO_2_, respectively). The weight loss of ~5 % around 100 °C can be attributed to the desorption of H_2_O molecules adsorbed in the Fe-N_*x*_C_*y*_/N-C catalysts, which matches well with the endothermic peaks in the DSC and a peak at 100 °C in the MS curves. The weight loss of ~25 % for Fe-N_4_ SAs/N-C and Fe-N_3_C_1_ SAs/N-C catalysts and ~10% for Fe-N_2_C_2_ SAs/N-C catalyst from 100 °C to 800 °C can be attributed to the desorption of adsorbed CO_2_ molecules and the decomposition of functional groups on carbon surface to form CO_2_, which respectively correspond to the two peaks at 250 °C and 550 °C in the MS curves. From the X-ray photoelectron spectroscopy (XPS) analysis of Fe 2*p* spectra (Supplementary Fig. 10a), a noticeable shift to lower binding energy of Fe 2*p*_3/2_ can be observed from Fe-N_4_ SAs/N-C to Fe-N_2_C_2_ SAs/N-C, revealing the gradually decreasing oxidation state of Fe. The negative shift from Fe-N_4_ SAs/N-C to Fe-N_2_C_2_ SAs/N-C in Fe 2*p* also indicates that the strong interaction between Fe and N atoms is decreased. From the N 1*s* spectra, three types of N species, namely, pyridinic N (398.3 eV), pyrrolic N (399.4 eV) and graphitic N (400.6 eV), can be distinguished in three Fe-N_*x*_C_*y*_ SAs/N-C catalysts (Supplementary Fig. 10b). Additionally, the pyridinic N contents decrease while the graphitic N contents increase gradually with the increase of pyrolysis temperature, which suggests that the Fe-N coordination number may vary. The Auger spectra (Supplementary Fig. 11) of three Fe-N_*x*_C_*y*_ SAs/N-C catalysts have some shift to the high electron energy from Fe-N_4_ SAs/N-C to Fe-N_2_C_2_ SAs/N-C, which indicates that the valence of Fe atom in Fe-N_4_ SAs/N-C is higher than that of Fe-N_3_C_1_ SAs/N-C and Fe-N_2_C_2_ SAs/N-C catalysts; this difference can be attributed to the different coordination environments, demonstrating the existence of coordination effect. Furthermore, the valence band spectra (Supplementary Fig. 12) of Fe-N_4_ SAs/N-C, Fe-N_3_C_1_ SAs/N-C, and Fe-N_2_C_2_ SAs/N-C catalysts were recorded by ultraviolet photoemission spectroscopy (UPS). It can be clearly seen that the valence band shifts away from the Fermi level in the order of Fe-N_2_C_2_ SAs/N-C, Fe-N_3_C_1_ SAs/N-C, Fe-N_4_ SAs/N-C, demonstrating that the *d* band center gradually changed^27,28^, which indicates that the interaction of Fe-N in Fe-N_4_ SAs/N-C makes more contribution to the valence band structure than does that of Fe-C in Fe-N_3_C_1_ SAs/N-C and Fe-N_2_C_2_ SAs/N-C catalysts.

X-ray absorption spectroscopy (XAS) analyses including X-ray absorption near-edge structure (XANES) and extended X-ray absorption fine structure (EXAFS) were conducted to systematically study electronic structures and coordination environments of the as-prepared Fe-N_*x*_C_*y*_ SAs/N-C samples. Soft XAS reveals that three peaks can be observed in C K-edge of all the Fe-N_*x*_C_*y*_ SAs/N-C catalysts (Fig. 2e). The peaks around about 285–286 eV and 293–295 eV are derived from sp^2^-hybridized carbon, assigned to the C-C π* and C-C σ* peaks, respectively^29^. The peak at about 287 eV can be attributed to C-N-C, revealing the existence of defect sites in carbon lattice. Compared with the C K-edge of the three samples, some interesting phenomena can be clearly found. From Fe-N_4_ SAs/N-C to Fe-N_2_C_2_ SAs/N-C, the C-C π* peaks shift towards higher energy, and the C-C σ* peak shifts towards lower energy; whereas the C-N-C peak does not shift, which indicates that the decrease of coordination number leads to the change of C-C lattice, while maintaining the C-N-C lattice. Additionally, we found that the intensity of the C-N-C peak decreases gradually due to the loss of N atoms at high pyrolysis temperature, implying that the coordination number may change. This phenomenon can also be observed in the N K-edge spectrum (Fig. 2f). The peaks a, b and c located around 398.4, 399.6, and 400.9 eV can be ascribed to π^*^-transition of the pyridinic N, pyrrolic N, and graphitic N, respectively. The peak d at 407–408 eV suggested the formation of C-N-C or C-N bond due to the excitations of σ*^30^. Similarly, with the decrease of coordination number, the intensities of peaks a and b also decrease gradually.

The XANES Fe L-edge spectra (Fig. 2g) of three samples show two typical L_3_ and L_2_ peaks at about 708 and 721 eV, respectively. The L-edge peak is due to the Fe electronic transitions from 2*p* orbitals to the unoccupied 3*d* orbitals^31^. Additionally, a negative shift for the L_3_ and L_2_ peaks can be observed from Fe-N_4_ SAs/N-C to Fe-N_2_C_2_ SAs/N-C catalysts, indicating elevated electron density around the Fe atoms^32,33^. The Fe K-edge XANES (Fig. 2h) indicates that the oxidation state of Fe in Fe-N_*x*_C_*y*_ SAs/N-C catalysts is between +2 (FeO) and +3 (Fe_2_O_3_). Moreover, a shift towards lower energy from Fe-N_4_ SAs/N-C to Fe-N_2_C_2_ SAs/N-C was observed, suggesting the decreased oxidation state of Fe^34^. The accurate coordination information of Fe-N_*x*_C_*y*_ SAs/N-C catalysts was verified by quantitative least-squares EXAFS curve-fitting analysis. The Fourier transform-EXAFS (FT-EXAFS) spectra of all the samples were first analyzed by using two backscattering paths of Fe-N and Fe-C and were found to have best-fitting results (Supplementary Fig. 13). Only a primary peak attributed to Fe-N(C) shell at R space of 1.4 Å can be observed (Fig. 2i), without the signal of Fe-Fe shell (compared with Fe foil), further indicating the atomic dispersion features of all the Fe atoms in Fe-N_*x*_C_*y*_ SAs/N-C catalysts. More importantly, the intensity of Fe-N shell in R space decreases with the increase of pyrolysis temperature, again demonstrating the decreased number of coordinating N atoms of Fe center. After EXAFS fitting (Supplementary Table 2), the coordination number can be directly obtained; the Fe-N coordination numbers in the first coordination shell are 4.2, 3.1, and 2.3, while the Fe-C coordination numbers are 0, 0.8 and 1.9 at the temperatures of 500, 600, and 700 °C, respectively. The wavelet transform (WT) contour plots (Fig. 2j) were carried out to further demonstrate the Fe-N pattern. We can see that among all the samples only one intensity maximum occurs at about 4 Å^−1^, without Fe-Fe signal (compared with Fe foil, FeO and Fe_2_O_3_). To further confirm the structure features, we performed the XANES simulations (solid lines) for the representative Fe-N_4_, Fe-N_3_C, and Fe-N_2_C_2_ structures using the ab initio multiple-scattering FEFF8.20 code^35^. To optimize the configuration for XANES simulations, we also constructed various models with different coordination geometries using DFT calculations (Supplementary Fig. 20). We calculated the XANES of each model and compared it with the experimental spectra of the catalysts (Fig. 2k–m). It turned out that the theoretically calculated spectra of Fe-N_4_, Fe-N_3_C, and Fe-N_2_C_2_ show similar features to the experimental ones, particularly for the shape and the position of the peak at about 7718 eV. All these results further demonstrate well-defined structure of the Fe-N_4_ SAs/N-C, Fe-N_3_C_1_ SAs/N-C, and Fe-N_2_C_2_ SAs/N-C catalysts, respectively.

### BOR performance

**Fig. 3:**
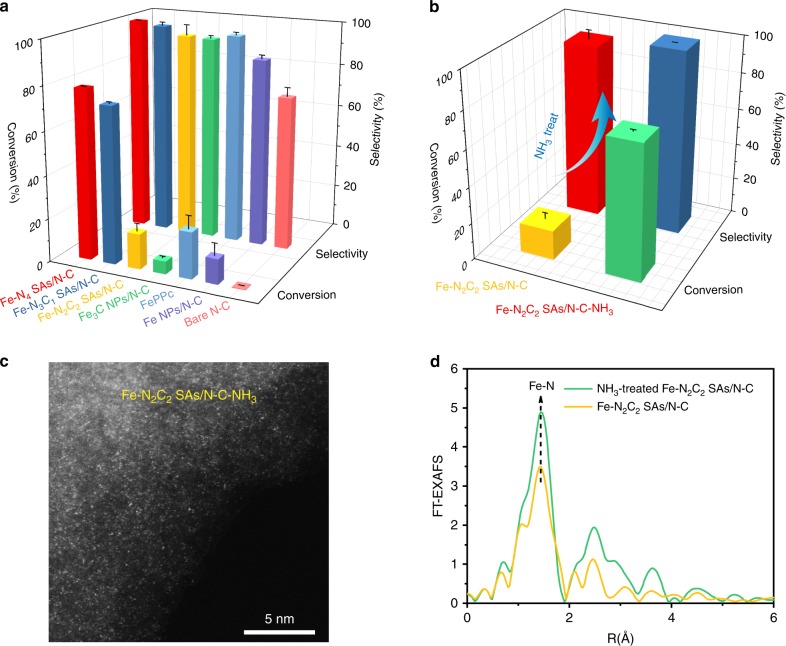
BOR performance and structure analysis. **a** Comparison of BOR performance of the as-synthesized different catalysts at 30 °C for 24 h. **b** Comparison of BOR performance of Fe-N_2_C_2_ SAs/N-C catalyst before and after NH_3_ treatment at 30 °C for 24 h. The error bars represent the standard deviation of three measurements. **c** AC-HAADF-STEM image of the Fe-N_2_C_2_ SAs/N-C-NH_3_. Scale bar, 5 nm. **d** FT k^3^-weighted χ(k)-function of the EXAFS spectra of the Fe-N_2_C_2_ SAs/N-C catalyst before and after NH_3_ treatment

The BOR performances of the as-prepared Fe-N_*x*_C_*y*_ SAs/N-C catalysts were evaluated at 30 °C. To our surprise, the Fe-N_*x*_C_*y*_ SAs/N-C catalysts exhibit excellent performance in catalytic oxidation of benzene to phenol with high benzene conversion and phenol selectivity (as shown in Fig. 3a). Additionally, the different Fe-N and Fe-C coordination environments lead to the different catalytic performances. With the increase of reaction time from 2 h to 24 h, the benzene conversion increased gradually and the phenol selectivity remains 100% (Supplementary Fig. 14). The optimized Fe-N_4_ SAs/N-C catalyst exhibits the highest performance for BOR with benzene conversion of 78.4% and a phenol selectivity of 100% within 24 h (Fig. 3a, Supplementary Fig. 14); with the reaction time further increased, the BOR performance of Fe-N_4_ SAs/N-C catalyst remained almost unchanged. Compared with reported BOR catalysts, the benzene conversion over our Fe-N_4_ SAs/N-C catalyst is the highest (Supplementary Table 3). The Fe-N_3_C_1_ SAs/N-C catalyst also shows the high benzene conversion. However, the conversion decreases significantly if the Fe-N coordination number is decreased to 2, suggesting that the coordination environment play an important role in BOR. In contrast, FePPc, Fe_3_C NPs/N-C, and Fe NPs/N-C shows poor BOR activity. It should be noted that the surface of carbon of all the catalysts may be oxidized to form surface functional groups during BOR, and therefore, the effect of surface chemistry of carbon on BOR performance was investigated by evaluating the BOR performance of bare N-C. We find that the bare N-C nearly has no activity, and thus, the effect of surface functional groups on BOR activity can be neglected.

To understand the adsorption capacity of O_2_ on the Fe-N_*x*_C_*y*_ SAs/N-C catalysts, we carried out the low-temperature O_2_ temperature-programmed desorption (TPD) measurement. As shown in the Supplementary Fig. 15, the TPD results indicate that, compared with Fe-N_3_C_1_ SAs/N-C and Fe-N_2_C_2_ SAs/N-C catalysts, the Fe-N_4_ SAs/N-C catalyst has a significantly higher adsorption capacity of O_2_, which is similar with previous studies^36,37^ reporting that the Fe-N_4_ structure in metal porphyrin or phthalocyanine is beneficial for oxygen adsorption, further revealing that the Fe-N_4_ SAs/N-C catalyst has more active sites than Fe-N_3_C_1_ SAs/N-C and Fe-N_2_C_2_ SAs/N-C catalysts.

The surface hydrophilicity/hydrophobicity of catalyst is also important factors to affect the adsorption and mass transfer of substrates, and would thus alter the catalytic performance. Therefore, we test the contact angle (CA) of the Fe-N_*x*_C_*y*_ SAs/N-C catalysts. As shown in Supplementary Fig. 16, the obtained CA are 135°, 140°, 146° for Fe-N_4_ SAs/N-C, Fe-N_3_C_1_ SAs/N-C, and Fe-N_2_C_2_ SAs/N-C catalyst, respectively. The Fe-N_4_ SAs/N-C catalyst shows the smallest CA, suggesting it has a better wettability, and the substrate molecules are easier to contact with catalyst, thereby improving the BOR performance.

**Fig. 4:**
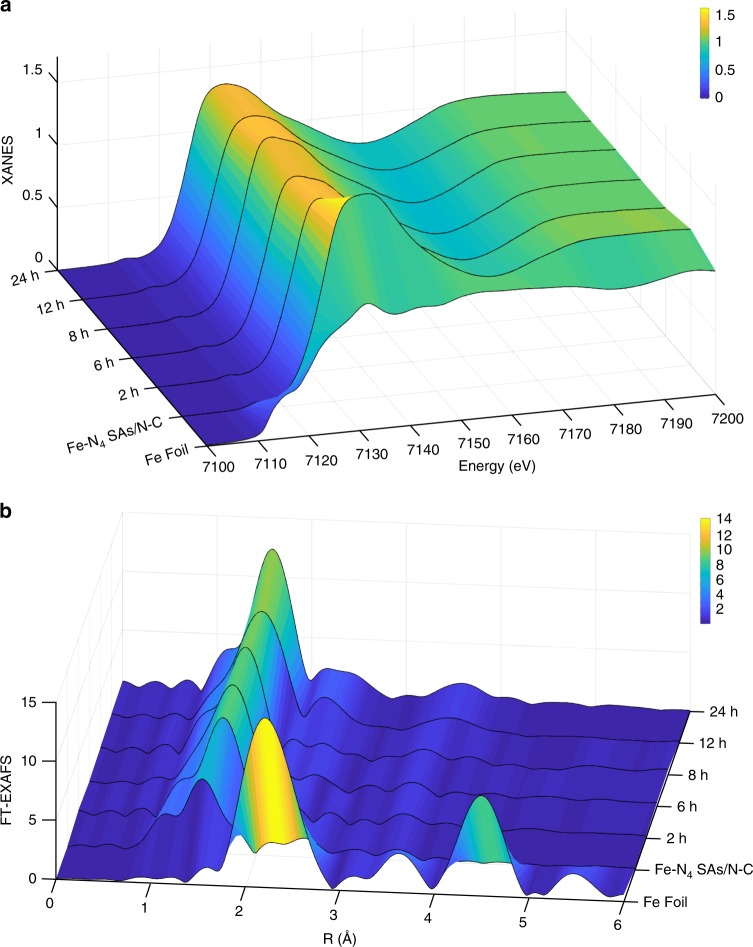
Stability investigations. **a**, **b** The Fe K-edge **a** XANES and **b** FT-EXAFS of Fe-N_4_ SAs/N-C catalyst at different BOR reaction time

In order to understand the coordination stability of isolated Fe-N_4_ species during BOR, we carried out the synchrotron radiation XAS measurement of Fe-N_4_ SAs/N-C catalyst at different BOR times to monitor the atomic structure evolution of Fe. As shown in the Fig. 4, With the BOR reaction time increased from 2 h to 24 h, nearly no obvious change can be found from the Fe K-edge XANES, indicating the high stability of the Fe near edge structure during BOR. The FT-EXAFS profiles at different BOR reaction times also show similar coordination environment except that the amplitude of the first shell peak is significantly enhanced and the bond length is increased compared with Fe-N_4_ SAs/N-C catalyst, which is due to the formation of Fe=O/O=Fe=O species on Fe-N_4_ species during BOR.

Consequently, we expected that, if the coordination number of Fe-N in Fe-N_2_C_2_ SAs/N-C catalyst is increased, the BOR performance should be enhanced. Therefore, we subjected the Fe-N_2_C_2_ SAs/N-C catalyst to NH_3_ at 500 °C for 3 h (denoted as Fe-N_2_C_2_ SAs/N-C-NH_3_). As expected, the catalytic activity of Fe-N_2_C_2_ SAs/N-C-NH_3_ catalyst is drastically enhanced with the benzene conversion elevated to 73.6 % (Fig. 3b). EDS mapping (Supplementary Fig. 17), AC HAADF-STEM (Fig. 3c), XANES and EXAFS (Fig. 3d, Supplementary Fig. 18) were used to further verify the atomic dispersion features, and the coordination number of Fe-N is found to be 3.5 ± 0.8 (Supplementary Fig. 19, Supplementary Table 2), which confirms our speculation. Element analysis result also indicates that the total N content is increased after the treatment of Fe-N_2_C_2_ SAs/N-C catalyst in NH_3_ (Supplementary Table 1).

### Intrinsic property of the Fe-N_*x*_C_*y*_ SAs/N-C catalysts

**Fig. 5:**
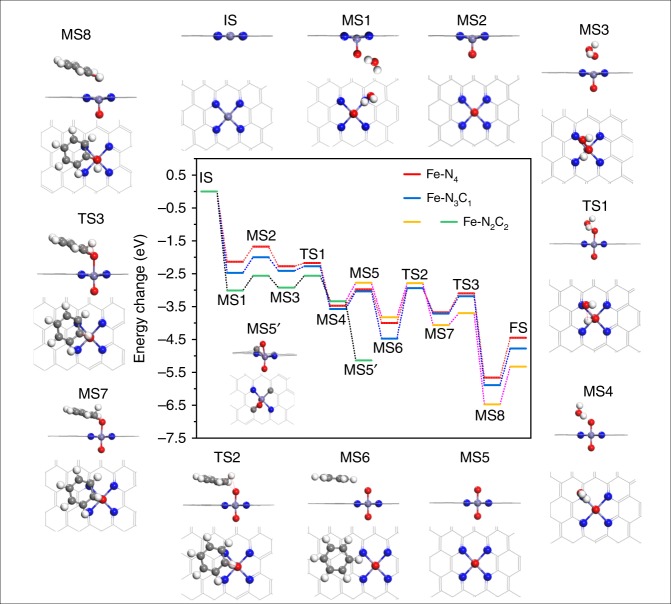
DFT calculation. Energy diagram of benzene oxidation on Fe-N_*x*_C_*y*_ SAs/N-C catalysts with the related reaction configurations on Fe-N_4_ surrounded (IS: initial catalyst, MS1: the first H_2_O_2_ cleavage adsorption, MS2: Fe=O configurations, MS3: the second H_2_O_2_ adsorption on the opposite side, TS1: transition state of the formation for the second H_2_O, MS4: the second H_2_O adsorbed configuration, MS5: O=Fe=O moiety, MS5′: the abnormal O=Fe=O species on Fe-N_2_C_2_, MS6: adsorption of C_6_H_6_, TS2: transition state of C–O bond generation, MS7: C_6_H_6_O adsorption structure, TS3: transition state of H transfer from C to O, MS8: product bonded species, FS: regeneration of activity Fe=O center). The white, gray, red, blue and bluish violet balls refer to H, C, O, N, and Fe atoms, respectively

To provide an in-depth understanding on the effect of coordination environment on the BOR activity, a systematic DFT study was carried out to investigate the electronic structure of the active site and reaction mechanism of the Fe-N_*x*_C_*y*_ SAs/N-C catalysts. The model in which the Fe-N_*x*_C_*y*_ site was embedded in a 6*6 cell of graphene was used (Supplementary Fig. 20). According to the optimized structures, the interactions between Fe and the neighboring N/C atoms are enhanced, with the Fe-N average distance (1.92 Å) shorter in Fe-N_2_C_2_ and Fe-N_3_C_1_ compared with the 1.95 Å Fe–N bond length in Fe-N_4_. At the same time, the Fe-C distances (1.90 Å and 1.87 Å, respectively in Fe-N_2_C_2_ and Fe-N_3_C_1_) are also rather short. The reaction minimum energy profile (MEP) of benzene oxidation on the specific iron site is shown in Fig. 5 (and the related data are presented in Supplementary Table 4). As is showed in the MEP, when approaching to the metal center the first H_2_O_2_ can dissociate easily and form a Fe = O intermediate (MS1) with the release of one H_2_O molecule. The Fe=O center (MS2) then serves as the active site for the oxidation of benzene. Inasmuch as M-N-C catalysts may exhibit enhanced reaction activity upon further axial coordination at heteroatoms and metal center of such catalysts^38,39^, the following reaction pathways on both Fe = O center are all taken into consideration. On the opposite Fe site, the second H_2_O_2_ molecule dissociated (TS1) with free energy barriers (ΔΔG1) of 0.07 eV, 0.11 eV, and 0.30 eV for Fe-N_4_, Fe-N_3_C_1_, and Fe-N_2_C_2_, respectively, forming O=Fe=O intermediates (MS5, Fig. 5 for Fe-N_4_, Supplementary Fig. 21 for both Fe-N_2_C_2_ and Fe-N_3_C_1_). The reaction continues via the C–O bond formation (TS2), with ΔΔG2 of 1.13 eV, 1.49 eV and −0.06 eV, and is finished by the transfer of hydrogen atom (TS3) from C to O and generation of phenol with ΔΔG3 of 0.56 eV, 0.44 eV, and 0.30 eV, respectively. Accordingly, the C–O bond formation step is the rate-limiting step. With the Fe=O center as a reference, the ΔΔG’ for C–O bond formation (TS2′) of Fe-N_3_C_1_ and Fe-N_4_ is 1.23 eV and 1.47 eV, and the C–O formation and H transfer achieve in one step for Fe-N_2_C_2_ (TS2”) with ΔΔG” of 2.16 eV (Supplementary Fig. 22, Supplementary Table 5).

The Fe-N_2_C_2_ catalyst with the O=Fe=O center shows excellent performance with the lowest barriers for both C–O bond formation and H transfer. However, during the formation of O=Fe=O moiety, there exists a low-energy intermediate (MS5′), in which the second oxygen is also strongly bonded with one of coordinated carbon atom, and the energy decrease as large as −2.36 eV relative to the normal O=Fe=O species on Fe-N_2_C_2_ (Supplementary Table 4). As a result, the Fe-N_2_C_2_ catalyst is deactivated by the generation of the O=Fe=O species. Overall, the Fe-N_4_ catalyst with a moderate free energy barrier and selectivity (the ΔΔG’ on Fe=O center is higher than that of ΔΔG2 on O=Fe=O when compared to Fe-N_3_C_1_) has well-balanced activity and selectivity. In other word, the oxidation of benzene on Fe-N_3_C_1_ might be obstructed due to the competition of related Fe=O intermediate. As for Fe-N_2_C_2_, the oxidation is difficult due to the O=Fe=O species.

**Fig. 6:**
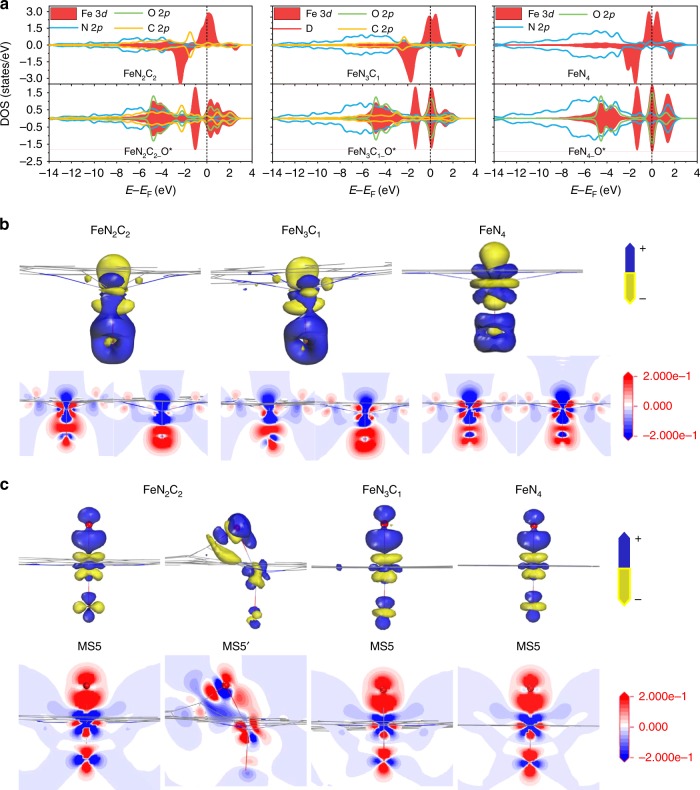
PDOS and charge density differences analyses. **a** PDOS for the metal center, atoms in the first coordinate shell and oxygen in Fe=O species (EF is marked in each graph with the black dash line). **b**, **c** Calculated charge density differences of **b** Fe=O and **c** O=Fe=O on Fe-N_*x*_C_*y*_ (*x* = 2, 3, 4 and related *y* = 2, 1, 0 from left to right) SAs/N-C catalysts (the characteristics of each color should be demarcated by the scale on the right)

The projected density of states (PDOS) for the metal center, atoms in the first coordinate shell and oxygen in Fe=O species were calculated (Fig. 6a). The PDOS also show obvious C 2p orbital contribution close to the Fermi energy level (EF) for both Fe-N_2_C_2_ and Fe-N_3_C_1_, suggesting that the interactions between two- and three-N doped graphene with Fe atom are stronger than that in Fe-N_4_. Additionally, the PDOS of Fe=O for Fe-N_2_C_2_ also shows an apparent charge transfer from Fe 3*d*-orbital to O 2*p*-orbital with the distribution of O 2*p* above the Fermi level. As a result, the Fe center in Fe-N_2_C_2_ is relatively charge-deficient, which tends to withdraw electron from graphene to the second oxygen, and leads to the strong C–O interaction in MS5′.

We further calculated the charge density differences of Fe=O on Fe-N_*x*_C_*y*_, as shown by three-dimensional (3D) map (upper) and two-dimensional (2D) profile (below) in Fig. 6b. In 2D profile of Fe=O, the three panels (from left to right), represent 2D profile along C-Fe-C, N-Fe-N planes of Fe-N_2_C_2_, C-Fe-N, N-Fe-N planes of Fe-N_3_C_1_ and N-Fe-N, N-Fe-N planes of Fe-N_4_, respectively. For single oxygen anchored structure Fe=O, Fe atom deviates from the plane of first coordinate shell with the distance of 0.49 Å, 0.42 Å and 0.30 Å, respectively, in Fe-N_2_C_2_, Fe-N_3_C_1_ and Fe-N_4_, and the related Fe-O distance of 1.60 Å, 1.62 Å, and 1.64 Å, respectively. The Fe=O bonds exhibit some covalent bond characteristic in C-containing profile, while in N-containing profiles, ionic bond characteristic and the lone pair electron property of O atom are more obvious, which indicate that the N-containing structure can promote the localization of O-site charge. Meanwhile, from the Fe-N_2_C_2_ to Fe-N_4_, the calculated Hirshfeld charge population of O atoms in Fe=O are −0.199 *e*, −0.212 *e* and −0.224 *e*, and the calculated binding energies of O atoms in Fe=O are −1.60 eV, −1.04 eV and −0.66 eV, respectively. These results suggest that the bonding strength between Fe-N_4_ and O atom is the weakest, while the charge transfer in it is the strongest, which may account for the highest catalytic activity.

The 3D map (upper) and 2D profile (below) of the charge density differences of O=Fe=O on Fe-N_*x*_C_*y*_, were shown Fig. 6c. In 2D profile of O=Fe=O, the three panels (from left to right), represent 2D profile along N-Fe-N plane in MS5, N-Fe-N plane in MS5′ of Fe-N_2_C_2_, N-Fe-N plane of Fe-N_3_C_1_ and N-Fe-N plane of Fe-N_4_, respectively. It can be seen that, in MS5 structure, the second O atom in Fe-N_*x*_C_*y*_ exhibits obvious lone-pair electron properties, and the charge density is highly concentrated at the O-site. However, in MS5′ structure of Fe-N_2_C_2_, there is a strong covalent characteristic in C–O, the charge density of O-site is decreased accordingly. Therefore, it can also be concluded that, with the decrease of Fe-N coordination number, the formation of O=Fe=O is not conducive, and the ability of activate C–H bond decreases accordingly. To sum up, the coordination pattern influences not only the structure and electronic features of the catalysts, but also the catalytic reaction pathway and the formation of each intermediate along the MEP.

In order to demonstrate the formation of Fe=O/O=Fe=O intermediates on Fe-N_4_ site, we carried out XAS, electron paramagnetic resonance (EPR), and ^57^Fe Mössbauer spectroscopy, respectively. Supplementary Fig. 23 shows the XANES and EXAFS of the Fe-N_4_ SAs/N-C catalyst after H_2_O_2_ treatment and BOR, it can be seen that the samples after the H_2_O_2_ treatment and BOR show similar XANES of the Fe K-edge with original Fe-N_4_ SAs/N-C catalyst, indicating the excellent stability of coordination geometry of isolated Fe species. We also note that the pre-edge peak (Fe 1*s*-to-3*d* transition) has been slightly broadened, which can be assigned to the formation of Fe=O which leads to Fe 3d mixing with O 2*p* and then destroys the Fe-N_4_ structured D_4h_ symmetry^40^. In addition, the FT-EXAFS also indicates that the amplitude of the first shell peak is significantly enhanced and the bond length is increased after the H_2_O_2_ treatment and BOR, which suggests that the coordination number of Fe species is increased, and we speculate that this may be due to the formation of Fe=O/O=Fe=O species during BOR.

We further used EPR (Supplementary Fig. 24) to characterize the BOR process in Fe-N_4_ SAs/N-C catalyst and after BOR at different times. It can be seen that the Fe-N_4_ SAs/N-C catalyst after BOR at 8 h, 12 h, and 24 h shows the obvious isotropic signal at *g* = 4.32, 4.38, and 4.46, respectively, which can be assigned to the Fe^IV^ = O species^8,38,41–44^ (which is considered to be the active intermediates of BOR), and the intensity increases with the increase of reaction time; however, the original Fe-N_4_ SAs/N-C catalyst shows very weak intensity. The enhanced EPR signals of Fe-N_4_ SAs/N-C catalyst after BOR at different times could be attributed to the probable formation of Fe=O/O=Fe=O.

To further support the above results, we used the ^57^Fe Mössbauer spectroscopy (Supplementary Fig. 25) to distinguish the change of Fe species in Fe-N_4_ SAs/N-C catalyst after the H_2_O_2_ treatment for 12 h and after BOR for 24 h. The Mössbauer fitting parameters and the relative areas of different Fe species were shown in Supplementary Table 6. It can be seen that the ^57^Fe Mössbauer spectra of three samples can be well-fitted with three doublets^38^. D1 represents the intermediate-spin Fe^II^-N_4_ species, D2 represents low-spin N-(Fe^III^N_4_)-N species, and D3 represents the high-spin adsorbed oxygen species on the both side of Fe-N_4_ SAs/N-C, respectively. We found that the relative areas of symmetrical O=Fe=O structure increases significantly in Fe-N_4_ SAs/N-C catalyst after the H_2_O_2_ treatment and BOR, revealing the formation of O=Fe=O species during BOR^7^. By combining the results of XAS, EPR, and ^57^Fe Mössbauer spectroscopy, we can conclude that the Fe-N_4_ site can effectively adsorb and activate H_2_O_2_ to produce O=Fe=O, which is an important active intermediate for catalyzing BOR.

## Discussion

In summary, a series of single-atom Fe sites with different Fe-N(C) coordination environments have been developed by an efficient PRP strategy. From both theoretical and experimental perspectives, we have shown that the N and C atoms in single-atom Fe-based catalysts play different roles in affecting the catalytic BOR activity. The single-atom Fe sites anchored by four-coordinating nitrogen atoms exhibit the highest BOR performance with a benzene conversion of 78.4% and a phenol selectivity of nearly 100%. When N atoms in Fe-N_4_ SAs/N-C catalyst are replaced by one or two C atoms, the activities decrease gradually, and the low activity also can be improved by increasing Fe-N coordination number. This result confirms that regulating the coordination environment of SACs can readily and efficiently change the catalytic performance. This work would provide deep understandings on the structure-activity relationship and catalytic mechanism at the atomic level, which could be helpful to develop advanced BOR catalysts and could also be extended to other catalytic applications.

## Methods

### Synthesis of FePPc and PPc

For the synthesis of FePPc, FeCl_3_ (0.486 g, 0.003 mol), urea (2.1 g, 0.035 mol), NH_4_Cl (0.5 g, 0.009 mol), (NH_4_)_2_Mo_2_O_7_ (0.013 g, 0.037 mmol), pyromellitic dianhydride (1.1 g, 0.005 mol) were mixed and ground uniformly in an agate mortar. Then the above mixture was transferred to a crucible and heated in a muffle furnace at 220 °C for 3 h with a ramp rate of 2 °C min^−1^. After cooling down to room temperature, the as-obtained product was washed many times using water, acetone, and methyl alcohol, respectively. Finally, the FePPc can be obtained after drying under vacuum at 60 °C for 12 h. Without the addition of metal salt, the PPc can be obtained.

### Synthesis of Fe-N_*x*_C_*y*_ SAs/N-C catalysts

For the synthesis of Fe-N_*x*_C_*y*_ with different coordination number, the powder of FePPc was placed in a tube furnace, kept at desired temperature (500 °C for Fe-N_4_ SAs/N-C, 600 °C for Fe-N_3_C_1_ SAs/N-C, 700 °C for Fe-N_2_C_2_ SAs/N-C) for 3 h with a heating rate of 2 °C min^−1^ under flowing Ar gas, and then naturally cooled to room temperature. The as-obtained samples were directly used without any post-treatment.

### NH_3_ treatment of Fe-N_2_C_2_ SAs/N-C

Typically, the as-synthesized Fe-N_2_C_2_ SAs/N-C was placed in a tube furnace, kept at 500 °C for 3 h with a heating rate of 2 ^o^C min^−1^ under flowing NH_3_ gas, and then naturally cooled to room temperature. The as-obtained samples were directly used without any post-treatment.

### Synthesis of Fe_3_C NPs/N-C and Fe NPs/N-C

The as-synthesized FePPc was placed in a tube furnace and heated to 800 °C with a ramp rate of 2 °C min^−1^ and kept for 3 h in flowing Ar, yielding Fe_3_C NPs/N-C. The obtained black powders were washed in 0.8 M H_2_SO_4_ solution at room temperature for 12 h, yielding Fe NPs/N-C.

### Synthesis of N-C

The as-synthesized PPc was placed in a tube furnace and heated to 900 °C with a ramp rate of 2 °C min^−1^ and kept for 3 h in flowing Ar to yield N-C.

### Catalytic test

The benzene oxidation reaction was carried out in a 50.0 mL glass reactor. 50 mg catalyst, 0.4 mL benzene, 6 mL H_2_O_2_ (30 wt%), and 3.0 mL CH_3_CN was added. After reaction at 30 °C for 24 h, 63.4 mg (0.28 mmol) n-hexadecane was added as the internal standard. After that, the mixture was extracted with ethyl acetate and centrifuged, then analyzed with gas chromatography (GC, Thermo Fisher scientific Trace 1300 with a flame ionization detector) and GC mass spectrometry (GC-MS, Thermo Fisher scientific ISQ system). The yield of phenol was calculated as (mole of formed phenol)/(mole of initial benzene) × 100%. The selectivity of phenol was calculated as (mole of formed phenol)/(mole of formed phenol + mole of formed benzoquinone) × 100%.

### Characterization

XRD was carried out with a Rigaku D/max 2500Pc X-ray powder diffractometer with monochromatized Cu Kα radiation (λ = 1.5418 Å). TEM was operated by a Hitachi-7700 working at 100 kV. HRTEM was carried out by a JEOL JEM-2100F field emission electron microscope working at 200 kV. AC-HAADF-STEM images were obtained by using a Titan 80–300 and Titan Cubed Themis 60–300 scanning/transmission electron microscope operated at 300 kV, equipped with a probe spherical aberration corrector. ICP-OES was carried out on Thermo Fisher IRIS Intrepid II. XPS was performed on a ULVAC PHI Quantera microscope. The binding energies (BE) were calibrated by setting the measured BE of C 1s to 284.8 eV. UPS was collected at the photoemission end-station at beamline BL11U in the National Synchrotron Radiation Laboratory (NSRL) in Hefei, China. The O_2_ TPD measurements were performed using a AutoChem II 2920 with a flowing 5% O_2_/He stream (50 mL min^−1^) at −50 °C. A Topologic 500 A spectrometer with a proportional counter was used to obtain the ^57^Fe Mössbauer spectroscopy. The radioactive source was ^57^Co(Rh), and α-Fe foil was used to calibrate the Doppler velocity of the spectrometer. MossWinn 3.0i program was used to fit the spectra with appropriate superpositions of Lorentzian lines. TG-MS was carried out using a NETZSCH STA449C (TG) coupled with TA-QMS 403C (MS) in an air atmosphere with a heating rate of 20 °C min^−1^. The CA of the catalysts was tested on Dataphysics DCAT21, Germany. EPR measurements were carried out on a Bruker E500 EPR spectrometry at 90 K. Elemental analysis was carried out on a PERKIN ELMER CE-440. Auger electron spectroscopy was carried out on a PHI 710 Scanning Auger Nanoprobe.

### XAFS measurements

The XAFS spectra were obtained at 1W1B station in BSRF (Beijing Synchrotron Radiation Facility, China) operated at 2.5 GeV with a maximum current of 250 mA. All samples were pelletized as disks of 13 mm diameter with 1 mm thickness using graphite powder as a binder. Additionally, the Fe L-edge, C K-edge and N K-edge X-ray absorption spectra were measured at beamline BL12B of National Synchrotron Radiation Laboratory (NSRL) of China and the samples were deposited onto double-sided carbon tap.

### XAFS analysis

The acquired EXAFS data were processed according to the standard procedures using the ATHENA module implemented in the IFEFFIT software packages. The EXAFS spectra were obtained by subtracting the post-edge background from the overall absorption and then normalizing with respect to the edge jump step. Then, χ(k) data in the k-space ranging from 2.6 to 12.6 Å^−1^ were Fourier transformed to real (R) space using hanning windows (d_k_ = 1.0 Å^−1^) to separate the EXAFS contributions from different coordination shells. The quantitative information can be obtained by the least-squares curve fitting in the R space with a Fourier transform k space range of 2.6 to 12.6 Å^−1^, using the module ARTEMIS of programs of IFEFFIT. The backscattering amplitude F(k) and phase shift Φ(k) were calculated using FEFF8.0 code.

The Fe K-edge theoretical XANES calculations were carried out with the MXAN code in the framework of multiple-scattering scheme using Muffin-tin approximation for the potential^45–48^. The self-energy dependent was calculated in the framework of the Hedin-Lundqvist scheme, and then the spectra was convoluted using a Lorentzian function with an energy-dependent width to account for the broadening due both to the core–hole width and to the final state width. In order to validate the reliability of the fitting procedure and the theoretical XANES spectrum calculation, all models were first optimized by DFT calculation. In the structure optimization, the four Fe-N/C distances were allowed to vary, and the optimized structure resulted in a distance of 1.93 Å between the iron and the four neighboring N/C atoms.

MXAN uses a phenomenological approach to calculate the inelastic losses on the basis of a convolution of the theoretical spectrum. Inelastic processes were taken into account by a convolution with a broadening Lorentzian function having an energy-dependent width of the form Γ (E) = Γ c + Γ mfp(E), in which the constant part, Γ c, takes care of both the core-hole lifetime and the experimental resolution, while the energy-dependent term represents intrinsic and extrinsic inelastic processes. The fitting quality was evaluated using the square residue function (R_sq_), where a statistical weight of 1 and a constant experimental error of 2.0% were used.

### DFT calculations

The density functional theoretical (DFT) calculations were performed by using the Perdew-Burke-Ernzerhof (PBE) functional within the formulation of generalized gradient approximation (GGA) as implemented in the DMOL^3^ program^49,50^. The Monkhorst-Pack scheme was used for sampling the Brillouin zone. The Fe-N_*x*_C_*y*_ models were set in a 6 × 6 supercell of graphene. The vacuum thickness between the catalyst slabs was set as 15 Å to get rid of the influence from the virtual interlayer interaction. The iron electrons are described by semi-core pseudopotentials (DSPPs) and a double numerical plus polarization (DNP) basis set, while the light element including C, O, N, H atoms are treated with all-electron basis sets. A Gaussian smearing finite-temperature broadening method (=0.005 Hatree) is used during structural optimizations. To ensure high-quality results, the real space atomic cutoff radius is chosen as 4.6 Å, and the Grimme parameters for van der Waals dispersion correction is also added to all calculations^51^. Kohn-Sham self-consistent field calculations are performed with convergence tolerance of 1 × 10^−6^ Hatree on the total energy. Vibrational frequency analysis was performed to gain the thermodynamic results. According to the vibrational analysis, the correlation of thermodynamic parameters (with the zero-point energy included) such as enthalpy (ΔH_corr_), entropy (ΔS_corr_) and free energy (ΔG_corr_) were taken into consideration in the study of the reaction mechanism. And the free energy at specific temperature were calculated by the formula G = *E*_total_ + G_corr_, where *E*_total_ is the total energy of the specific moiety on the MEP and G_corr_ is the free-energy correlation with the zero-point energy included at the specific temperature. So, the related free energy change at 298.15 K in each step was obtained by using the equation Δ*G* *=* Δ*E*_total_ +  ΔG_corr_, where Δ*E*_total_ is the energy difference of the total energy between each species, and ΔG_corr_ is the energy difference of the free energy correlations at 298.15 K.

## Supplementary information

Supplementary Information

## Data Availability

The data that support the findings of this study are available from the corresponding author upon reasonable request.
