# Supplementary material for: Regulating the coordination structure of single-atom Fe-NxCy catalytic sites for benzene oxidation
Source: Nat Commun. 2019 Sep 19;10:4290. doi: 10.1038/s41467-019-12362-8 (PMC6753116; doi:10.1038/s41467-019-12362-8)
Supplement: Supplementary file 1 — Supplementary Information [file 41467_2019_12362_MOESM1_ESM.pdf]

# Supplementary Information

## Regulating the coordination structure of single-atom Fe-$N_xC_y$ catalytic sites for benzene oxidation

**Pan et al.**

## Supplementary Figures

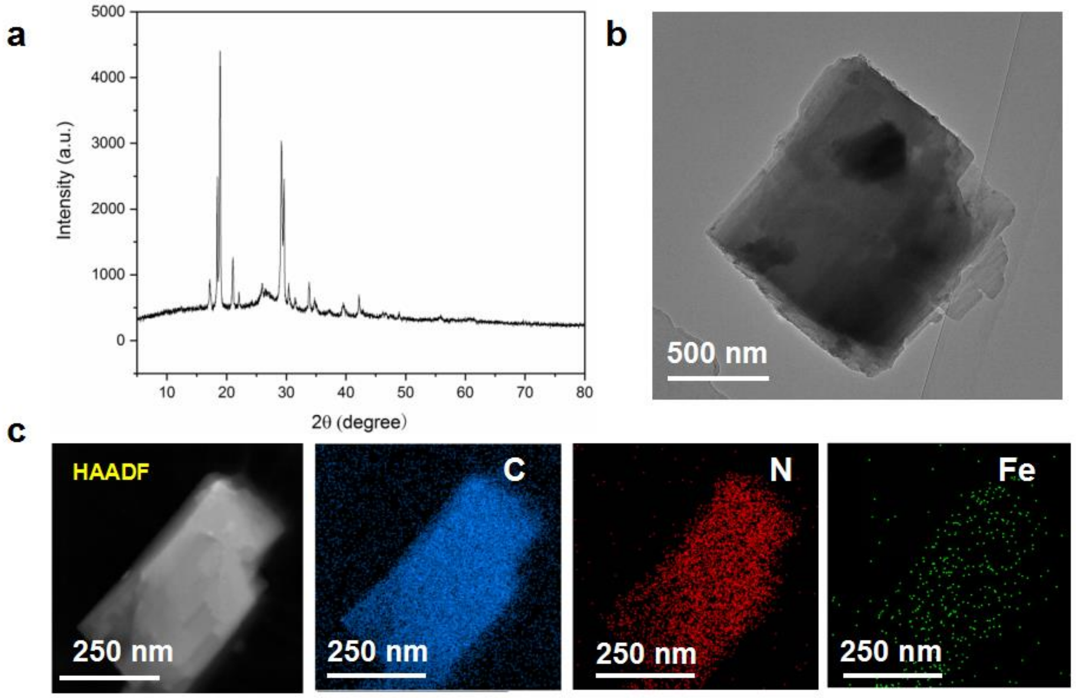


**Supplementary Figure 1. Structure characterizations of FePPc precursor.** (**a**) XRD pattern, (**b**) TEM image, (**c**) HAADF-STEM-EDS mapping images of the FePPc precursor.

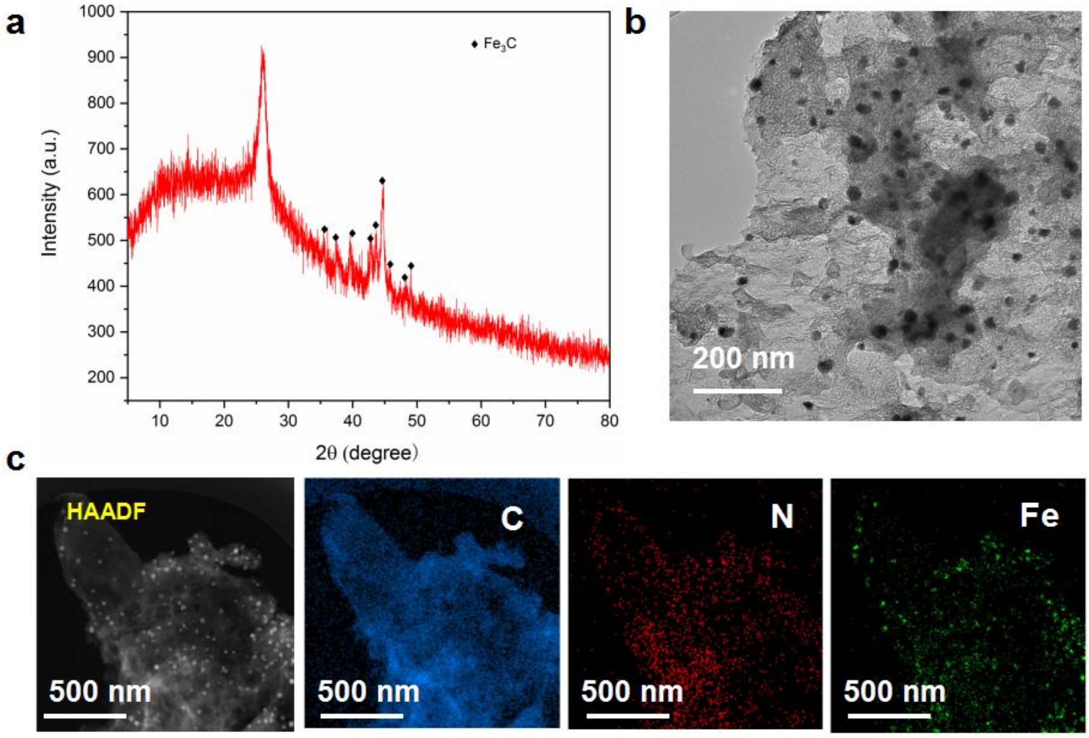


**Supplementary Figure 2. Structure characterizations of $Fe_3C$ NPs/N-C.** (**a**) XRD pattern, (**b**) TEM image, (**c**) HAADF-STEM-EDS mapping images of the sample obtained after pyrolysis of FePPc precursor at 800 ºC.

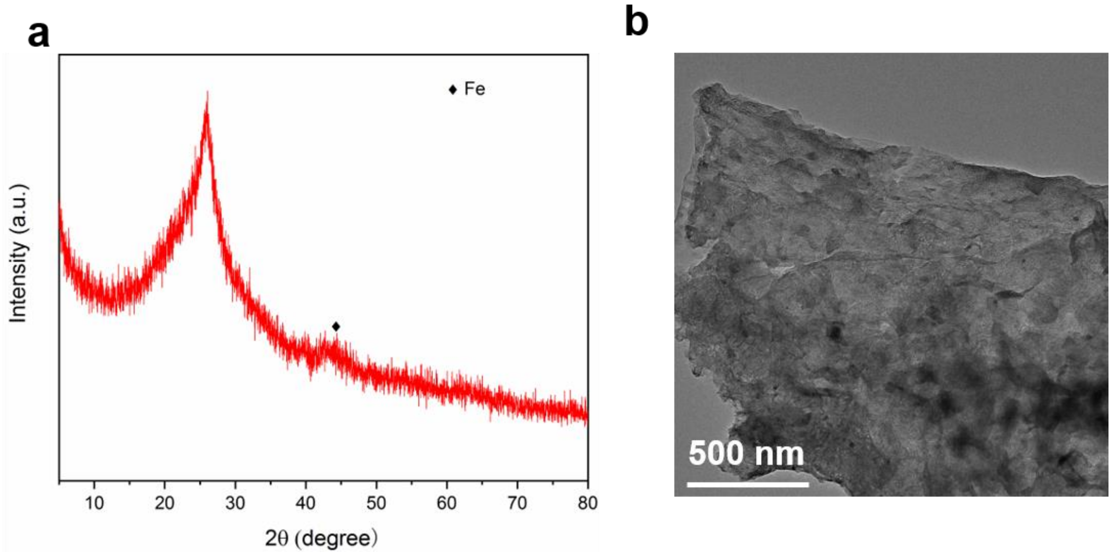


**Supplementary Figure 3. Structure characterizations of Fe NPs/N-C.** (**a**) XRD pattern and (**b**) TEM image of the sample obtained after pyrolysis of FePPc precursor at 800 ºC and then etching in sulphuric acid.

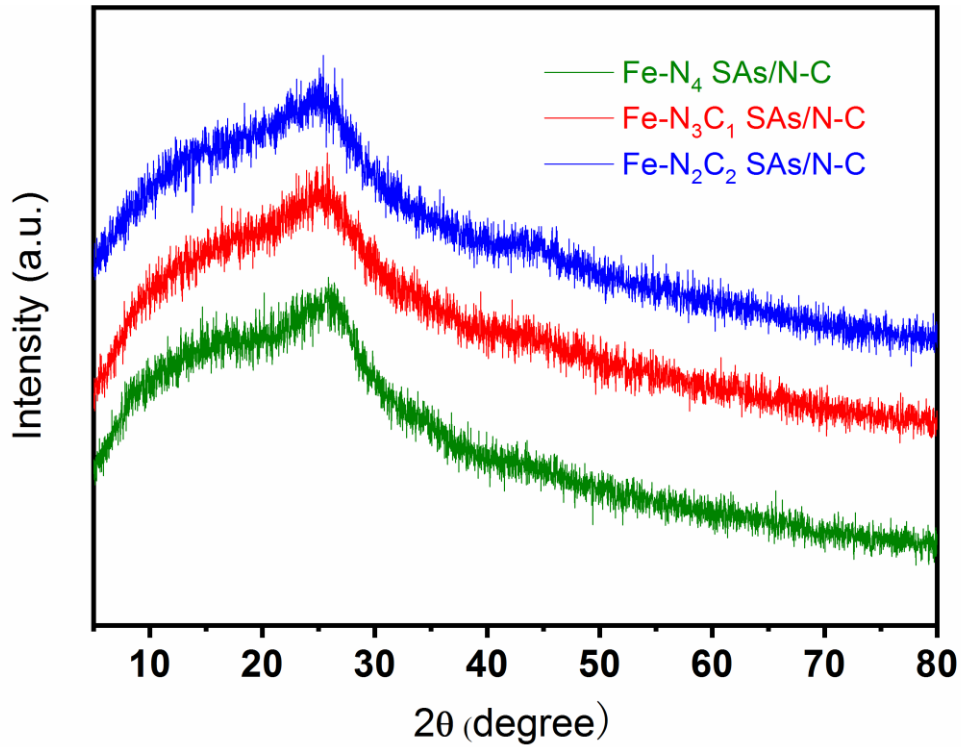


**Supplementary Figure 4. XRD characterizations of Fe-N$_x$C$_y$ SAs/N-C.** XRD patterns of the as-synthesized Fe-N$_x$C$_y$ SAs/N-C catalysts at different pyrolysis temperatures (500 ºC for Fe-$N_4$ SAs/N-C, 600 ºC for Fe-$N_3C_1$ SAs/N-C and 700 ºC for Fe-$N_2C_2$ SAs/N-C).

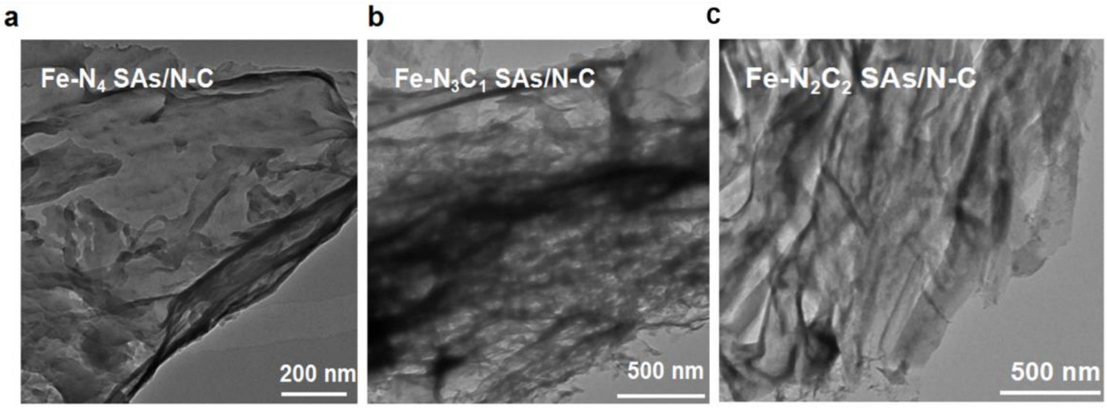


**Supplementary Figure 5. TEM characterizations of Fe-$N_xC_y$ SAs/N-C.** TEM images of the Fe-$N_xC_y$ SAs/N-C catalysts at different pyrolysis temperatures (500 ºC for Fe-$N_4$ SAs/N-C, 600 ºC for Fe-$N_3C_1$ SAs/N-C and 700 ºC for Fe-$N_2C_2$ SAs/N-C).

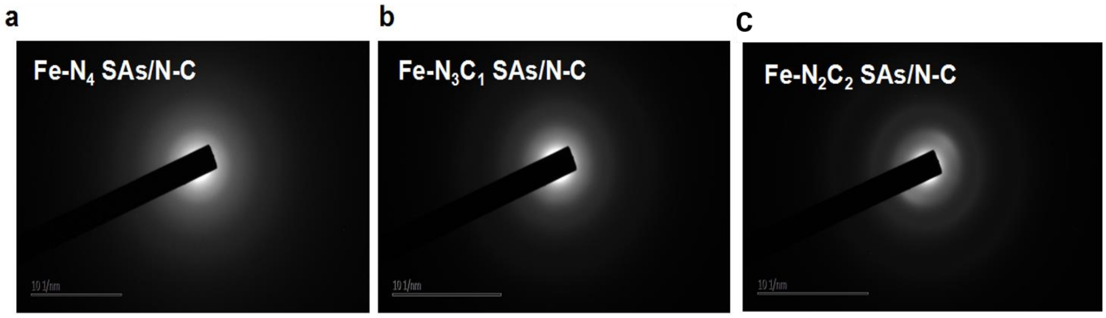


**Supplementary Figure 6. SAED characterizations of Fe-$N_xC_y$ SAs/N-C.** SAED images of the Fe-$N_xC_y$ SAs/N-C catalysts at different pyrolysis temperatures. (**a**) 500 ºC for Fe-$N_4$ SAs/N-C, (**b**) 600 ºC for Fe-$N_3C_1$ SAs/N-C and (**c**) 700 ºC for Fe-$N_2C_2$ SAs/N-C).

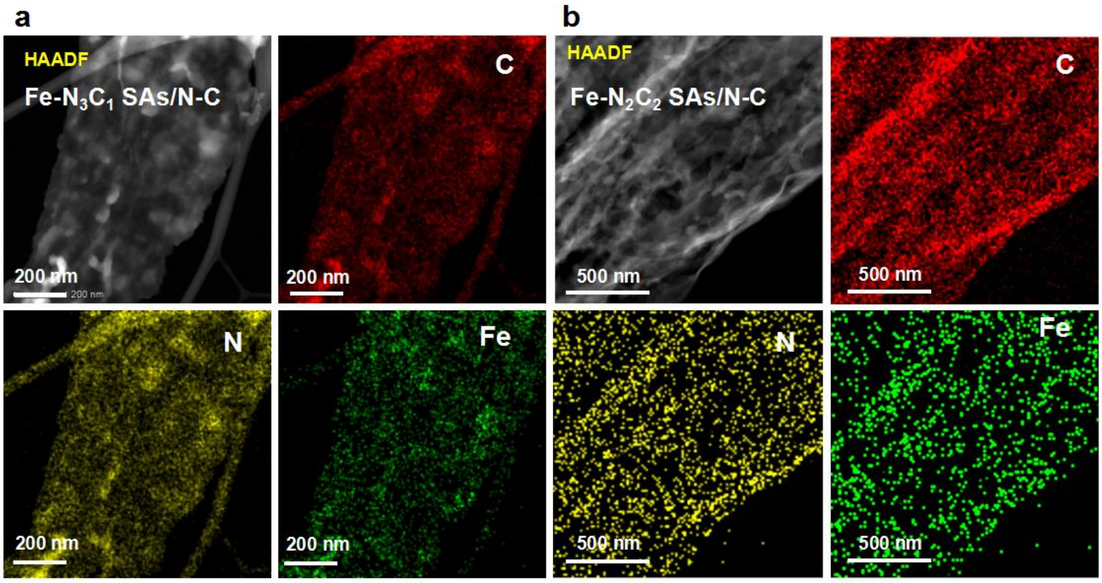


**Supplementary Figure 7. HAADF-STEM-EDS characterizations of Fe-$N_xC_y$ SAs/N-C.** HAADF-STEM-EDS mapping images of the (**a**) Fe-$N_3C_1$ SAs/N-C and (**b**) Fe-$N_2C_2$ SAs/N-C catalysts.

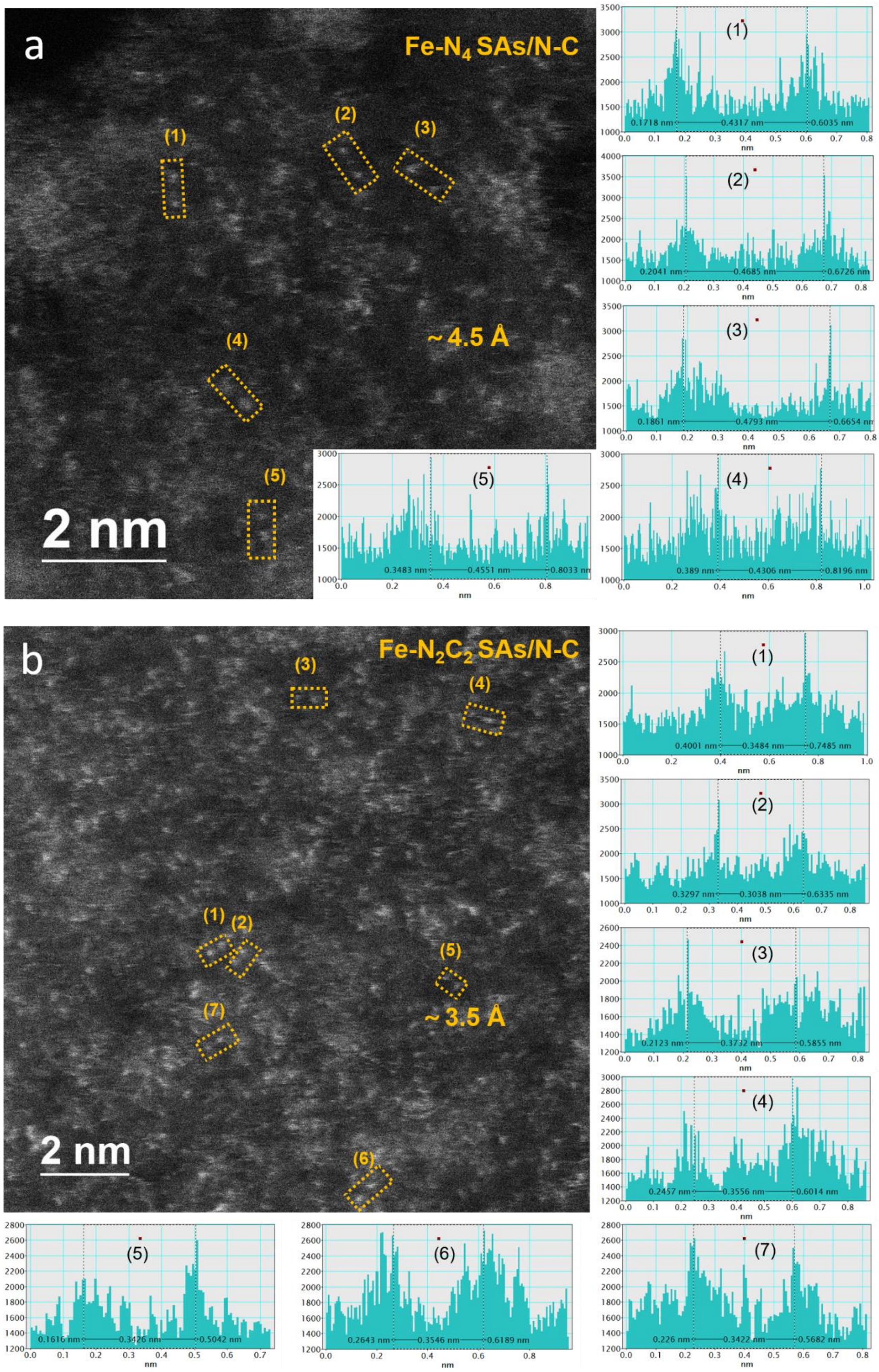


**Supplementary Figure 8. AC-HAADF-STEM characterizations of Fe-N$_x$C$_y$ SAs/N-C.** AC-HAADF-STEM image and the intensity profiles obtained in areas labeled 1-5 in (**a**) for Fe-$N_4$ SAs /N-C sample and labeled 1-7 in (**b**) for Fe-$N_2C_2$ SAs/N-C sample, respectively.

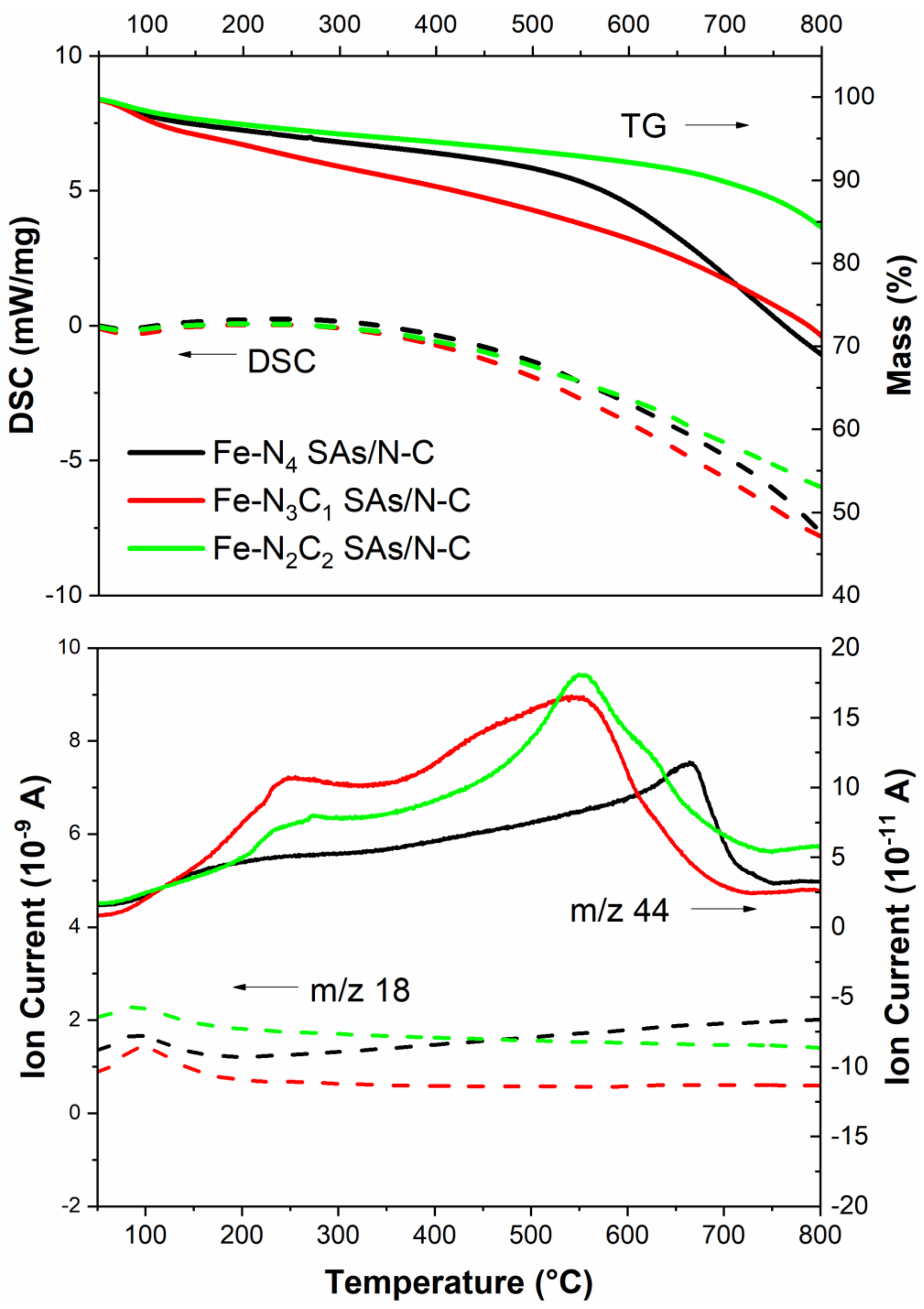


**Supplementary Figure 9. TG-DSC characterizations of Fe-$N_xC_y$ SAs/N-C.** TG-DSC curves and the corresponding MS curves of the Fe-$N_4$ SAs/N-C, Fe-$N_3C_1$ SAs/N-C, and Fe-$N_2C_2$ SAs/N-C catalysts.

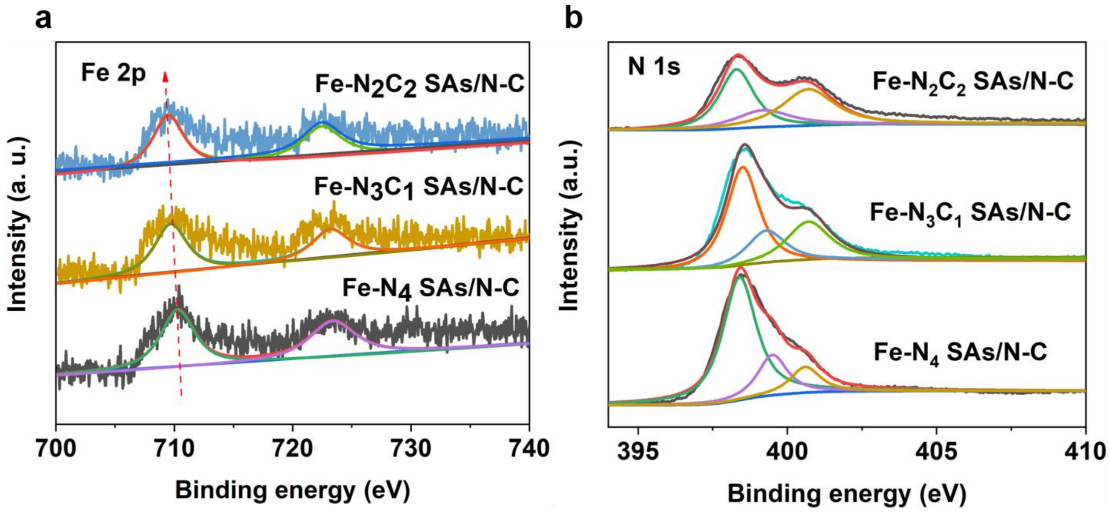


**Supplementary Figure 10. XPS characterizations of Fe-N$_x$C$_y$ SAs/N-C.** XPS spectra of (a) Fe 2*p* and (b) N 1*s* for Fe-$N_4$ SAs/N-C, Fe-$N_3C_1$ SAs/N-C and Fe-$N_2C_2$ SAs/N-C catalysts.

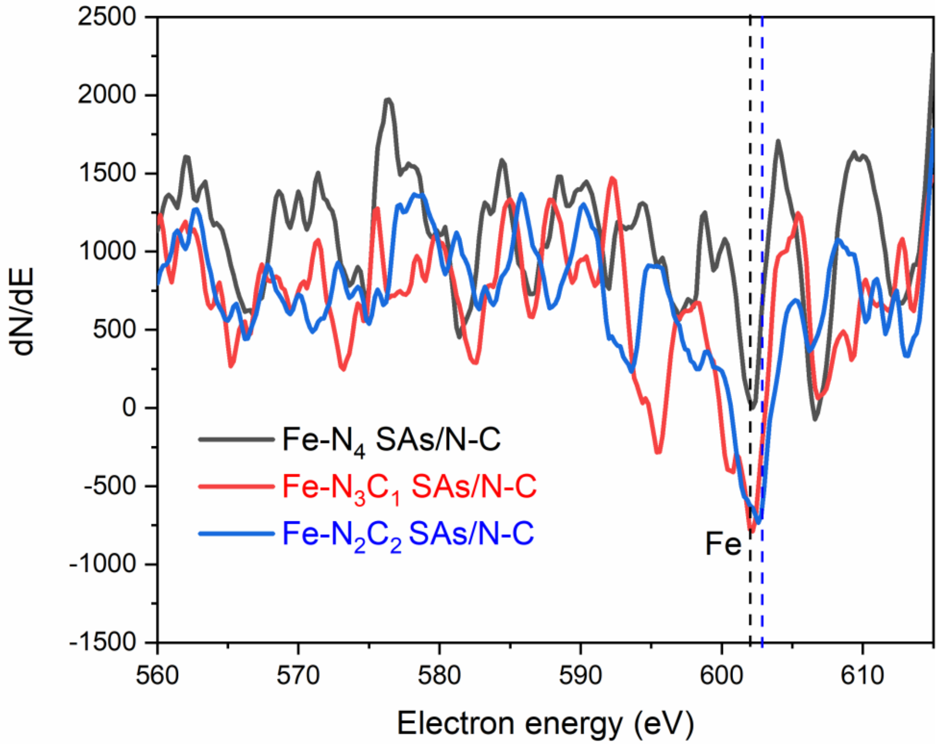


**Supplementary Figure 11. Auger spectra characterizations of Fe-$N_xC_y$ SAs/N-C.** Auger spectra of Fe-$N_2C_2$ SAs/N-C, Fe-$N_3C_1$ SAs/N-C, and Fe-$N_4$ SAs/N-C, respectively.

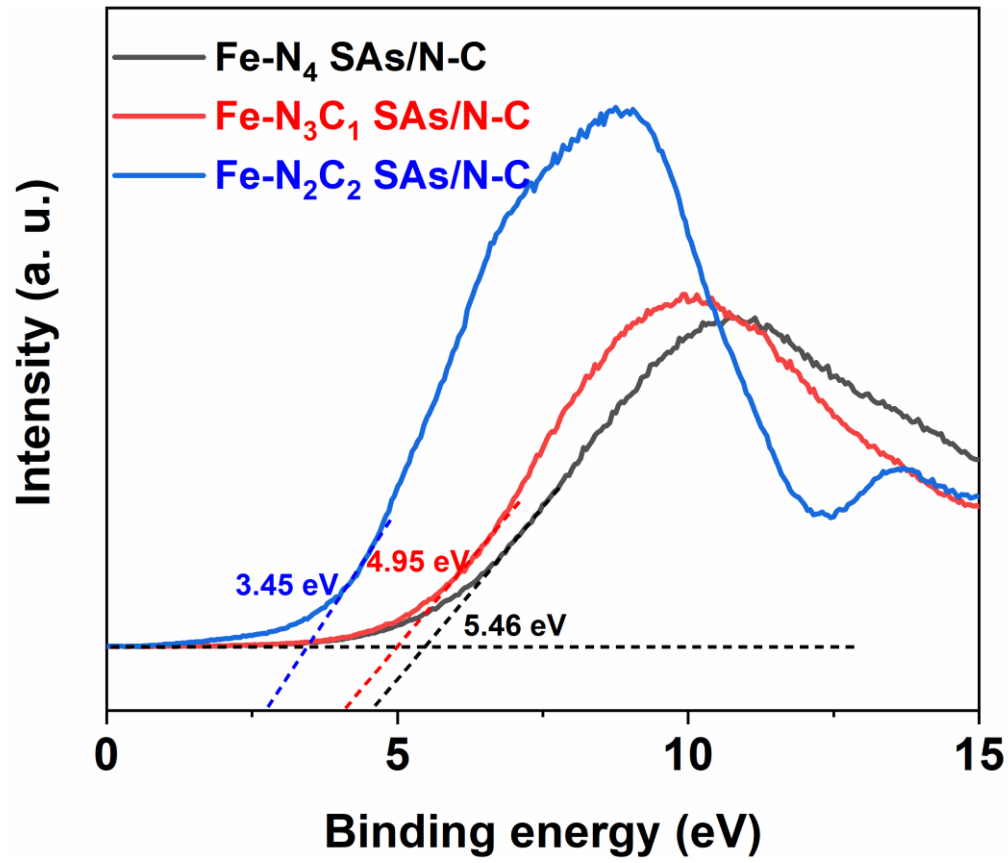


**Supplementary Figure 12. UPS characterizations of Fe-N$_x$C$_y$ SAs/N-C.** UPS curves of the Fe-$N_4$ SAs/N-C, Fe-$N_3C_1$ SAs/N-C, and Fe-$N_2C_2$ SAs/N-C catalysts.

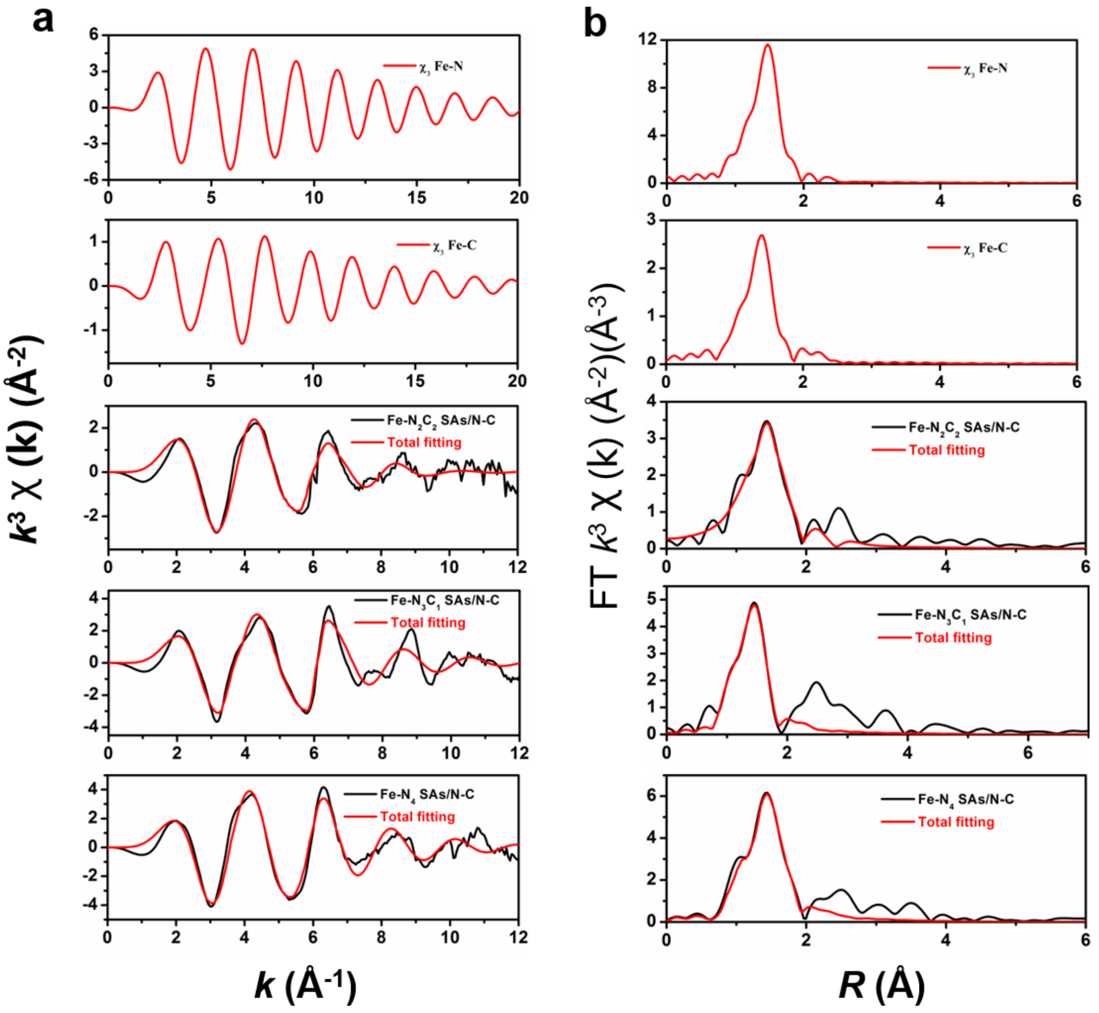


**Supplementary Figure 13. Fe K-edge EXAFS analysis of Fe-$N_4$ SAs/N-C, Fe-$N_3C_1$ SAs/N-C and Fe-$N_2C_2$ SAs/N-C in k (a) and R (b) spaces.** Curves from top to bottom are the Fe-N, Fe-C and two-body backscattering signals $\chi^2$ included in the fit and the total signal (red line) superimposed on the experimental signal (black line). The measured and calculated spectra are in good agreement.

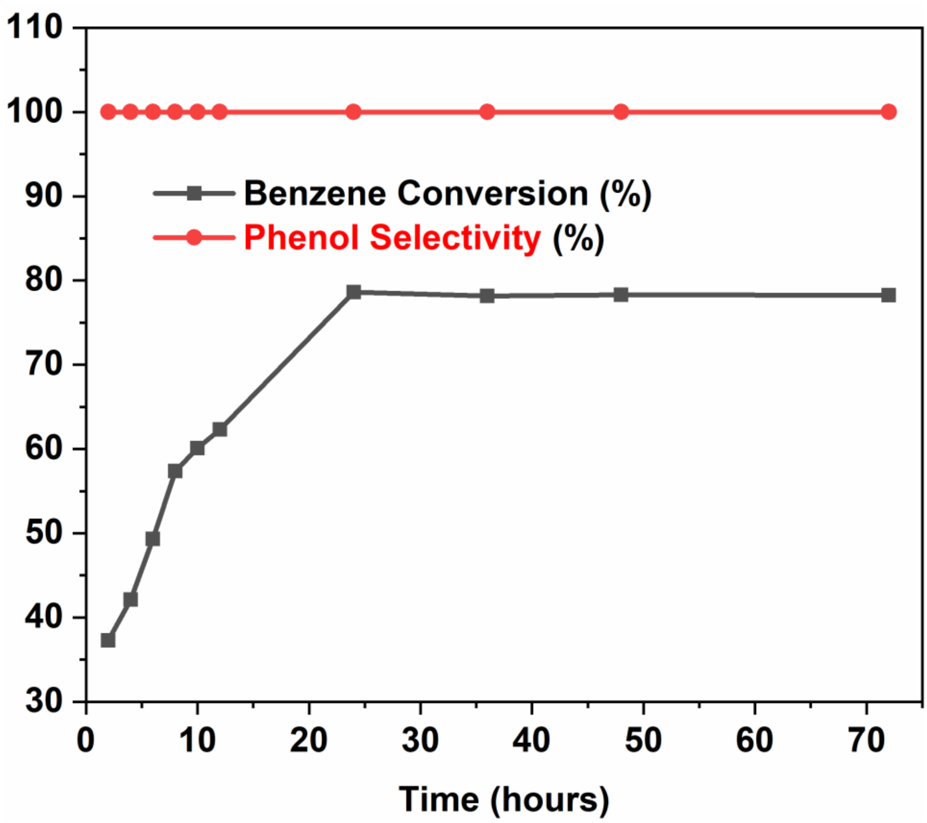


**Supplementary Figure 14. BOR performance of the Fe-$N_4$ SAs/N-C catalyst at different time.** The benzene conversion and phenol selectivity of Fe-$N_4$ SAs/N-C catalyst for the direct oxidation of benzene to phenol with different reaction times.

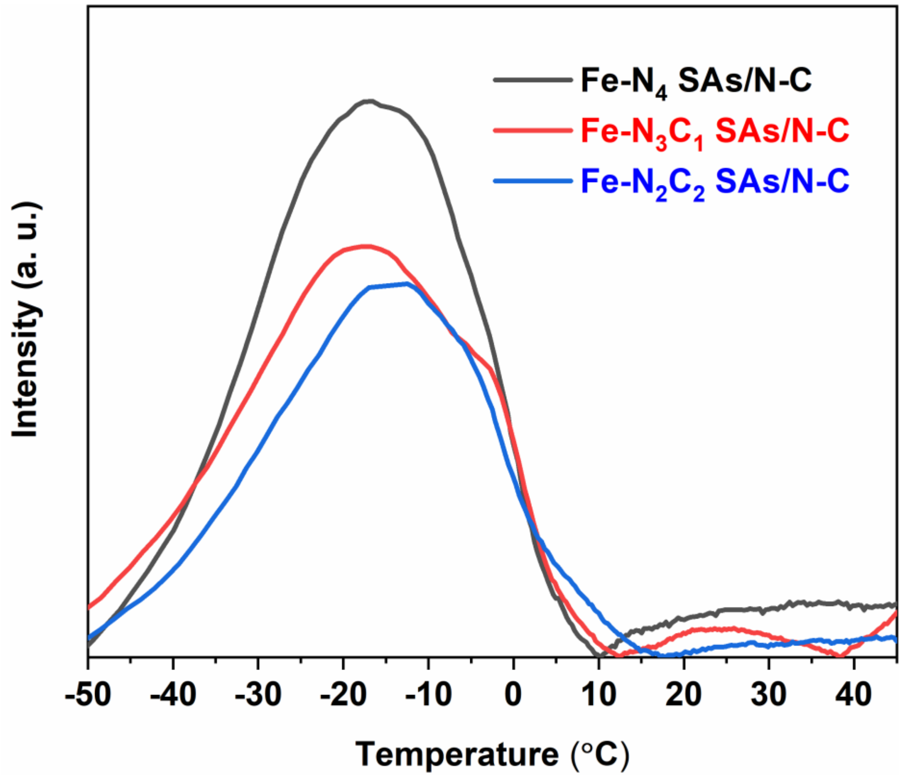


**Supplementary Figure 15. $O_2$ TPD characterizations of Fe-N$_x$C$_y$ SAs/N-C.** Low-temperature $O_2$ TPD profiles of the Fe-$N_4$ SAs/N-C, Fe-$N_3C_1$ SAs/N-C, and Fe-$N_2C_2$ SAs/N-C catalysts.

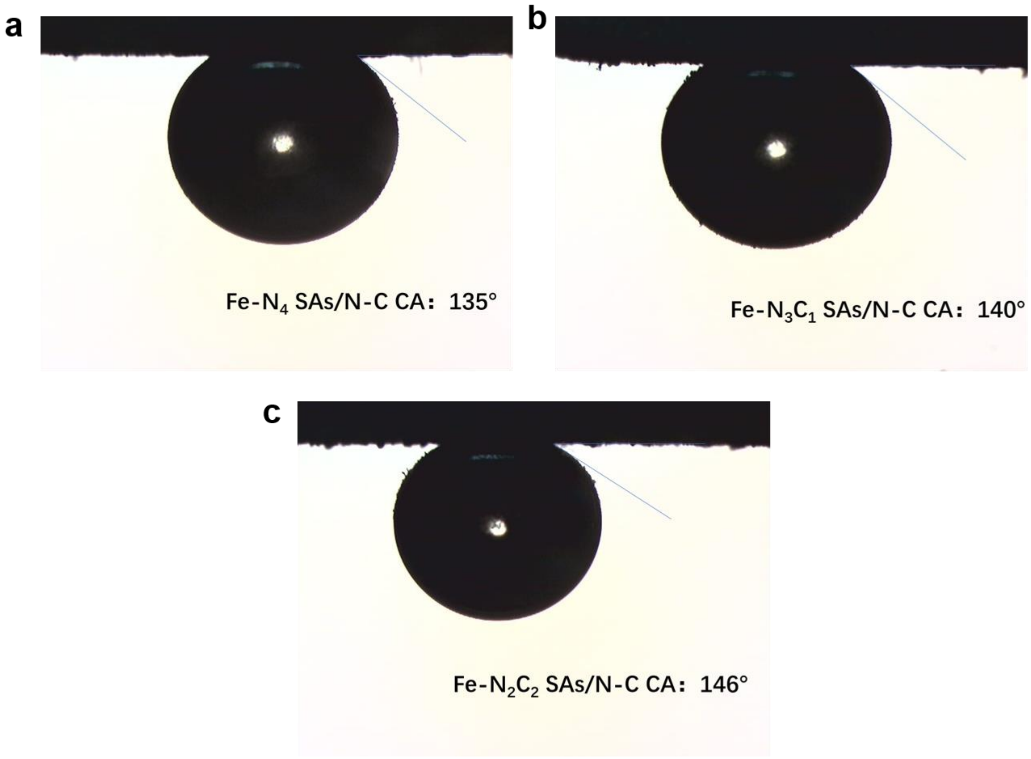


**Supplementary Figure 16. CA measurements of Fe-$N_xC_y$ SAs/N-C.** CA of the (**a**) Fe-$N_4$ SAs/N-C, (**b**) Fe-$N_3C_1$ SAs/N-C, and (**c**) Fe-$N_2C_2$ SAs/N-C catalysts.

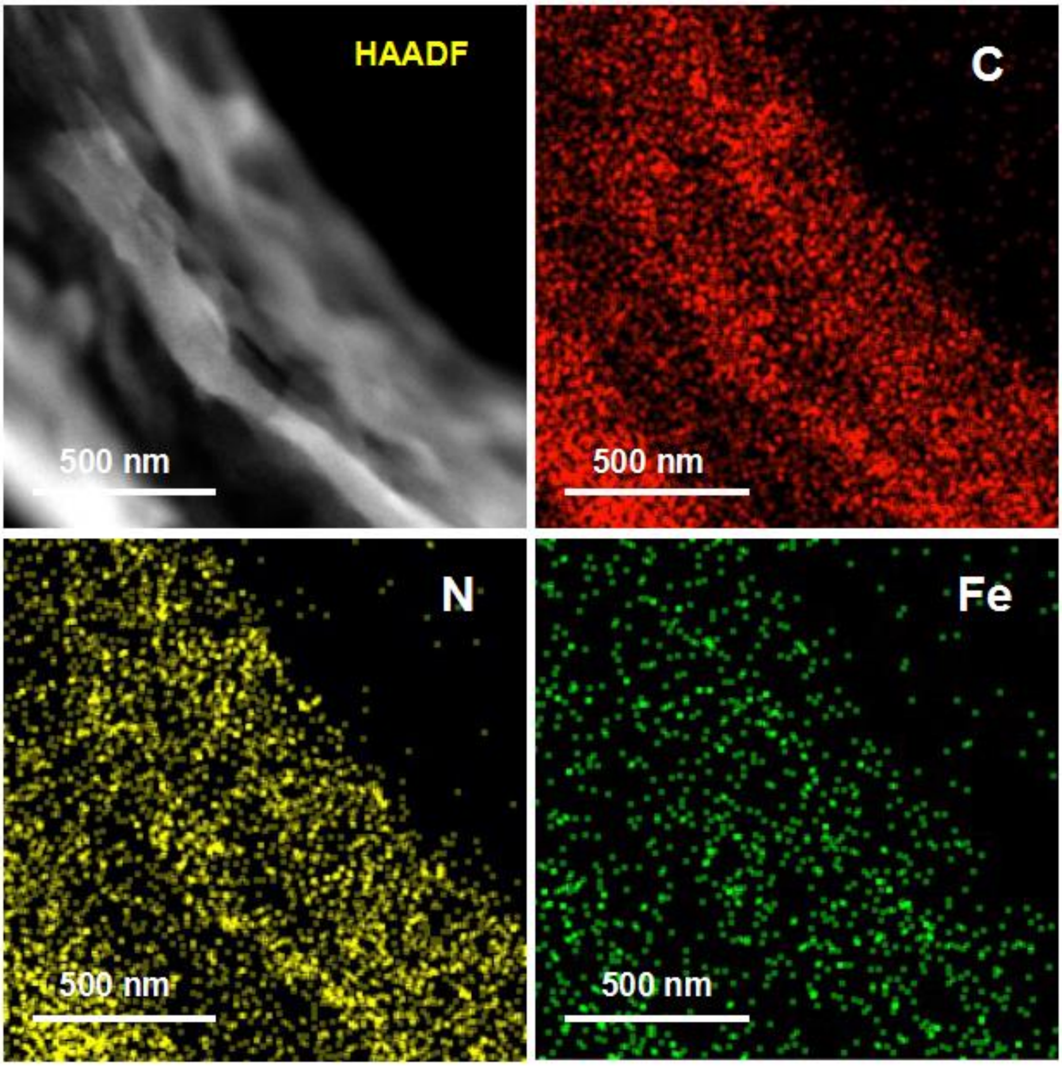


**Supplementary Figure 17. HAADF-STEM-EDS mapping images of the Fe-$N_2C_2$ SAs/N-C catalyst after $NH_3$ treatment.**

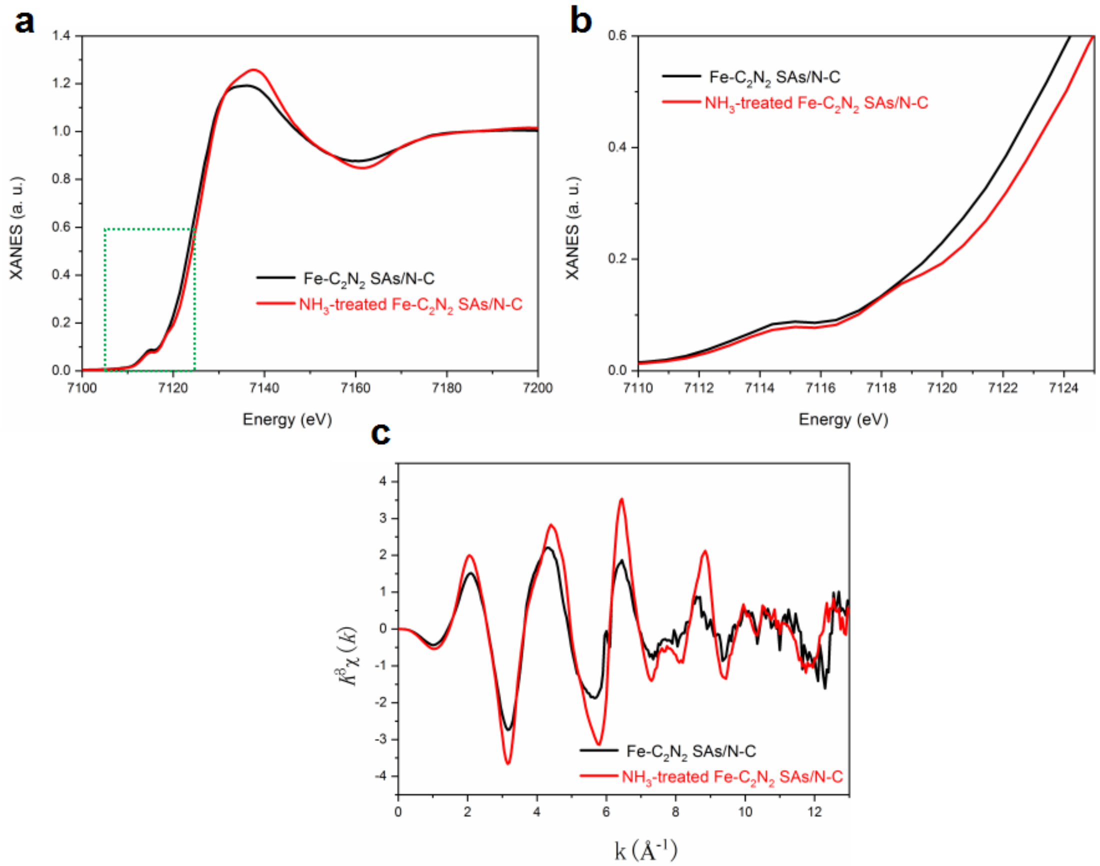


**Supplementary Figure 18. XAS characterizations of Fe-$N_2C_2$ SAs/N-C before and after $NH_3$ treatment.** (**a**) XANES spectra at the Fe K-edge, (**b**) the magnified image and (**c**) EXAFS in k space of the Fe-$N_2C_2$ SAs/N-C and $NH_3$-treated Fe-$N_2C_2$ SAs/N-C catalysts. From the Supplementary Figure 16(**b**), it can be seen that a noticeable shift to higher energy after Fe-$N_2C_2$ SAs/N-C was treated by $NH_3$, which indicated that the Fe oxidation state was increased, revealing the change of Fe coordination environment.

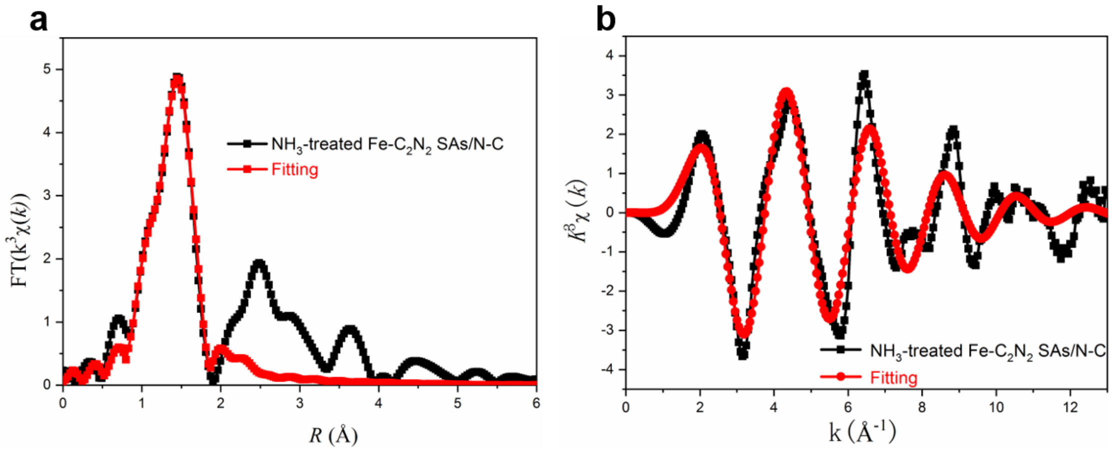


**Supplementary Figure 19. EXAFS fitting.** (**a**) EXAFS in k space of $NH_3$-treated Fe-$N_2C_2$ SAs/N-C catalyst. (**b**) The corresponding EXAFS fitting of $NH_3$-treated Fe-$N_2C_2$ SAs/N-C catalyst at R space.

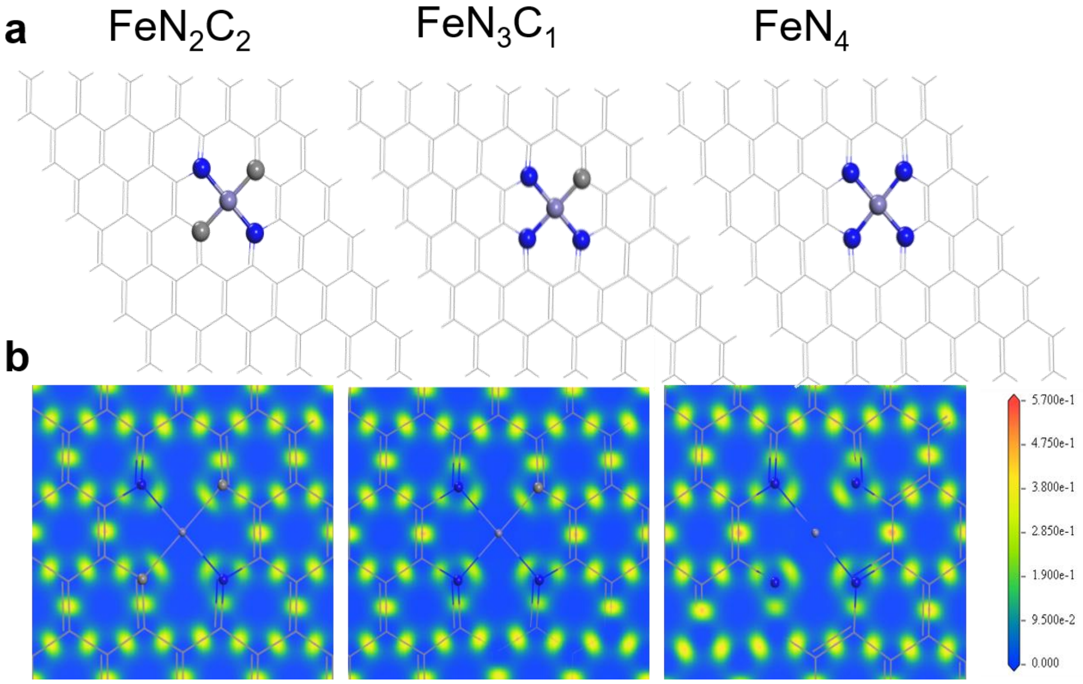


**Supplementary Figure 20. Computational models and ELF.** (**a**) Computational models used for the Fe-$N_xC_y$ SAs/N-C catalysts and **(b**) the corresponding ELF maps.

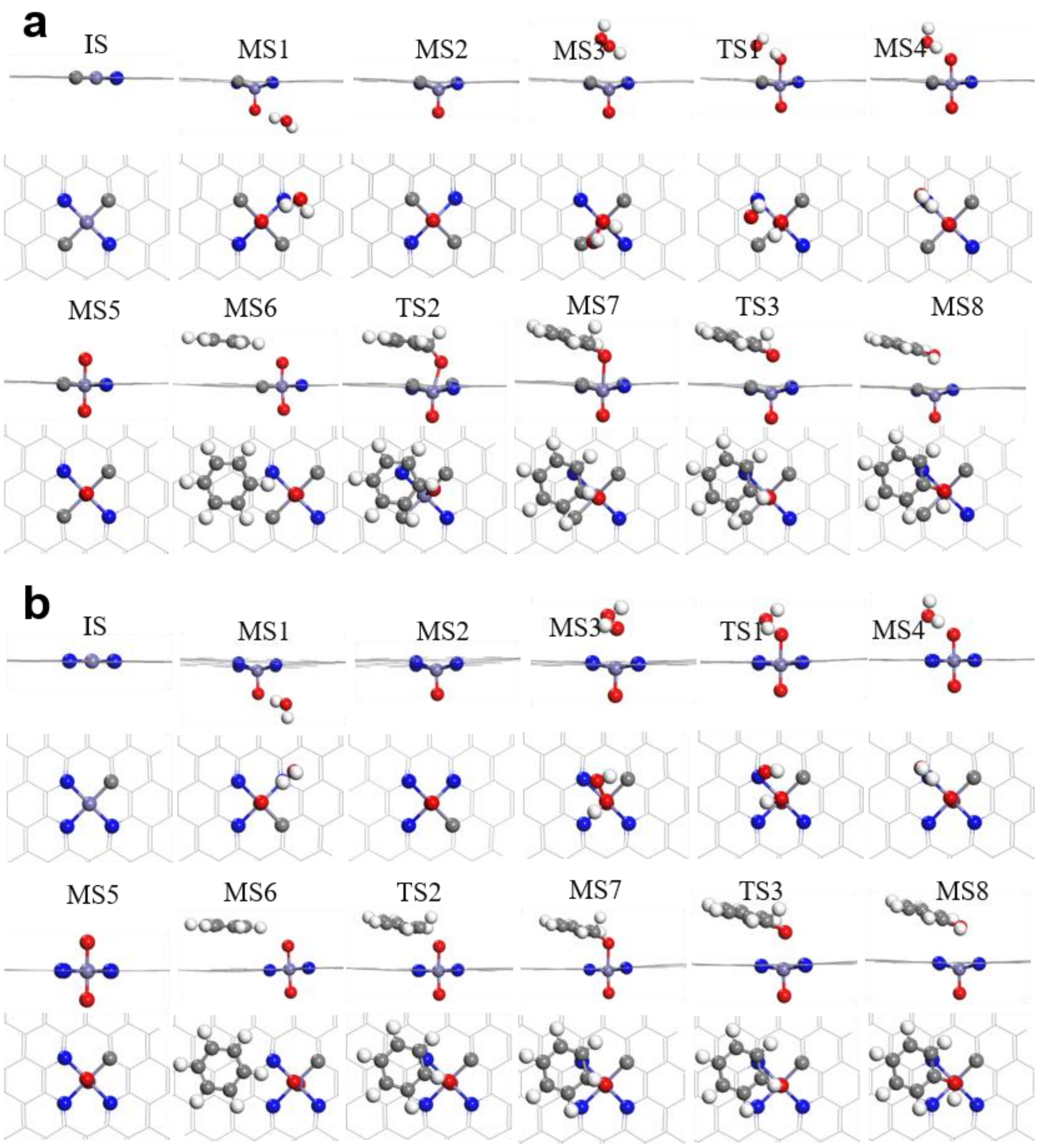


**Supplementary Figure 21. Structural configurations.** Structural configurations along the reaction energy diagram in the sequence of IS, MS1, MS2, MS3, TS1, MS4, MS5, MS6, TS2, MS7, TS3, MS8 on the (**a**) Fe-$N_2C_2$ SAs/N-C and (**b**) Fe-$N_3C_1$ SAs/N-C catalysts (from left to right and the top two rows to the bottom two row, side view and top side alternately presented in adjacent rows).

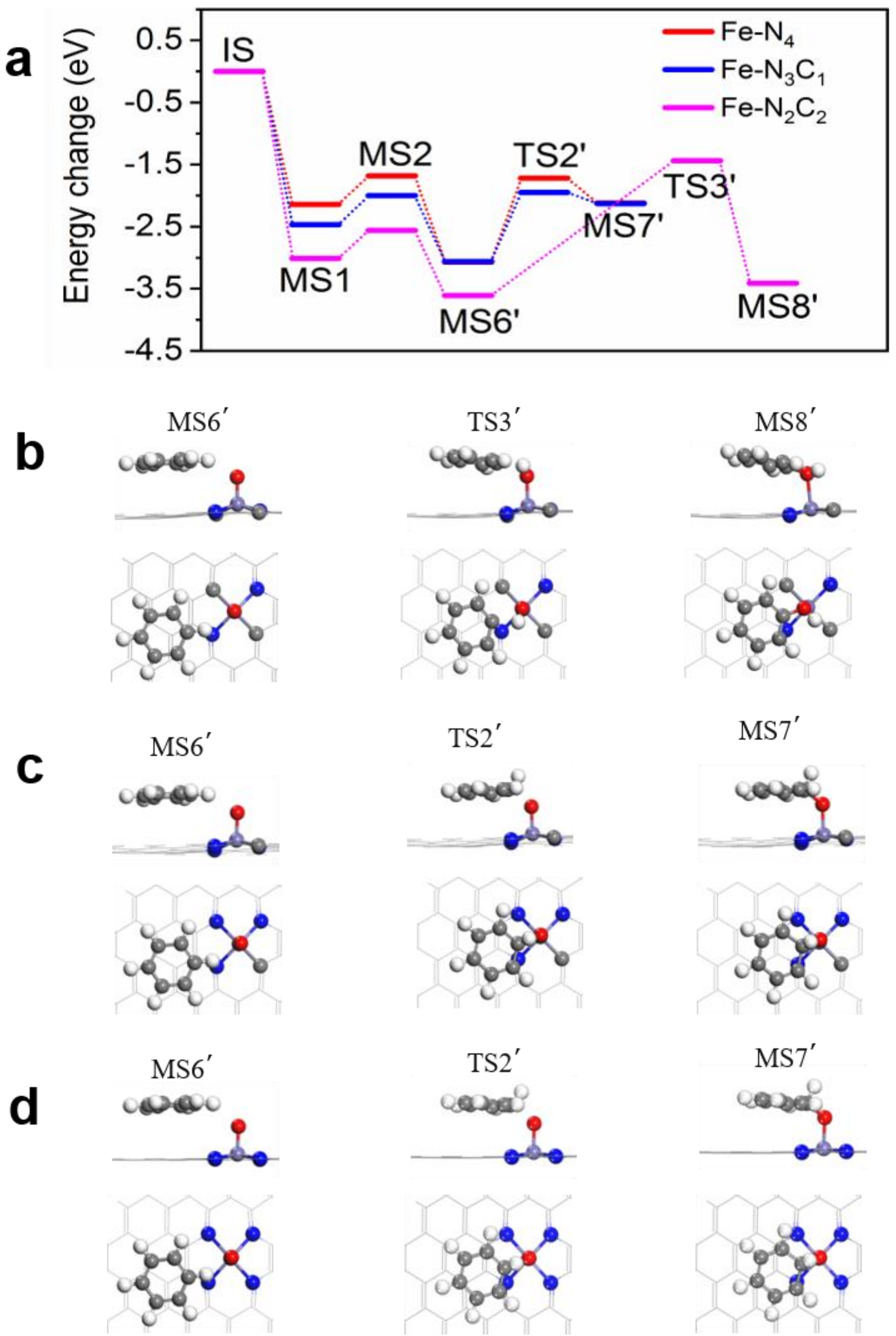


**Supplementary Figure 22. DFT calculation.** (a) Energy diagram of benzene oxidation on Fe=O species (MS6′: benzene adsorbed on Fe=O, TS2′: transition state of C-O bond generation, MS7′: $C_6H_6O$ adsorption structure, TS3′: transition state of the H transfer form C to O, MS7′: product bonded species); (b) related reaction configurations on Fe=O intermediate for (b) Fe-$N_2C_2$ SAs/N-C and (c) Fe-$N_3C_1$ SAs/N-C and (d) Fe-$N_4$ SAs/N-C catalysts.

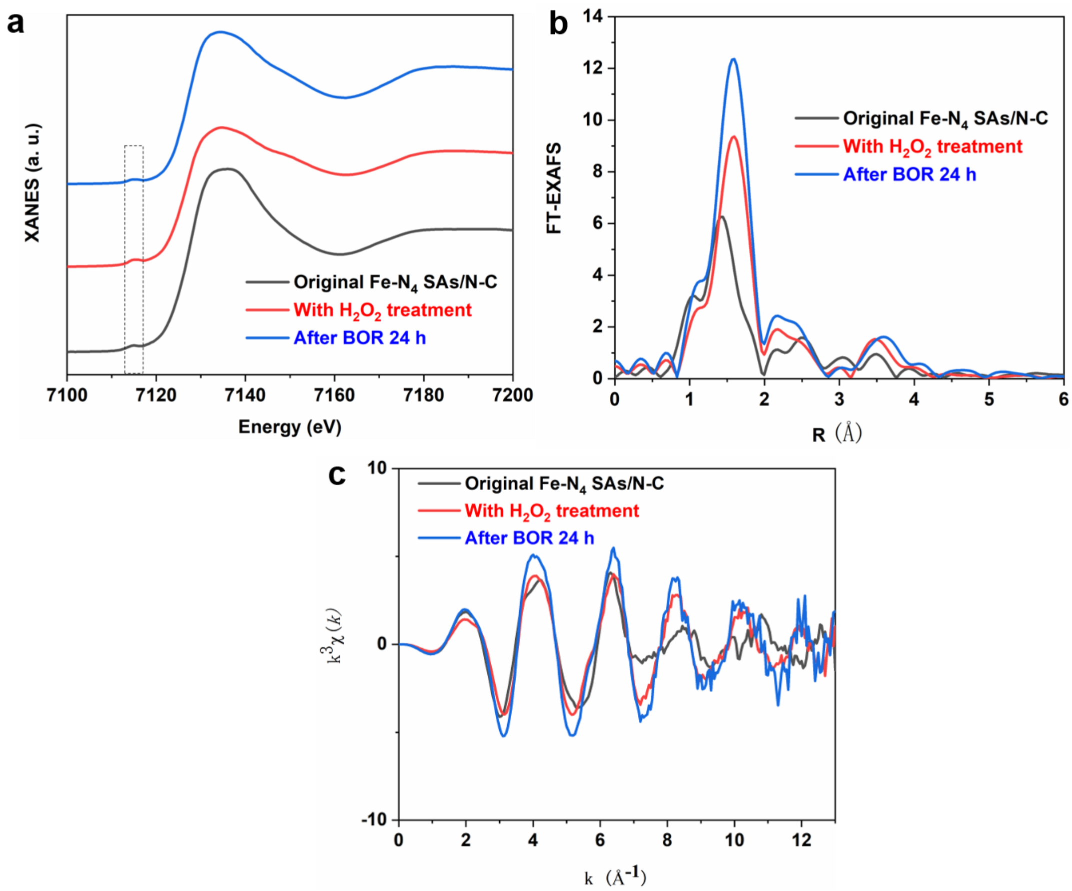


**Supplementary Figure 23. XAS characterizations of $Fe-N_4$ SAs/N-C before and with $H_2O_2$ treatment and after BOR.** The Fe K-edge (a) XANES, (b) FT-EXAFS, and (c) k space of the $Fe-N_4$ SAs/N-C catalyst after $H_2O_2$ treatment for 12 h and after BOR for 24 h in comparison to the original sample.

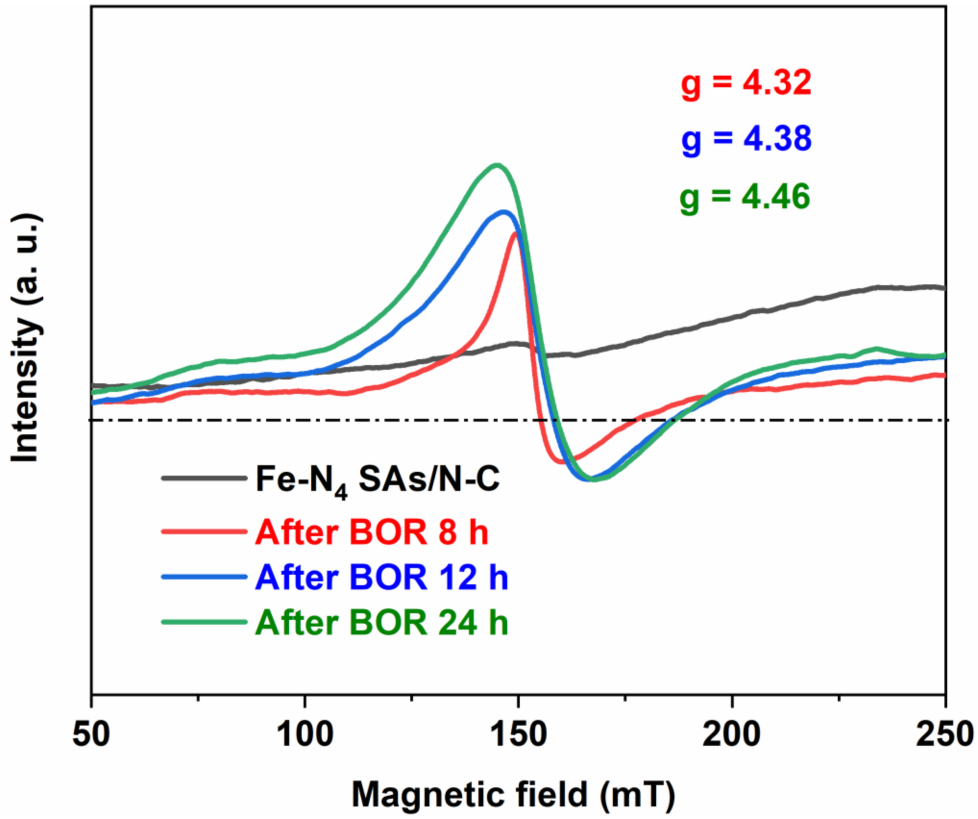


**Supplementary Figure 24. EPR characterizations**. EPR spectra of the Fe-$N_4$ SAs/N-C catalyst and after BOR at different times.

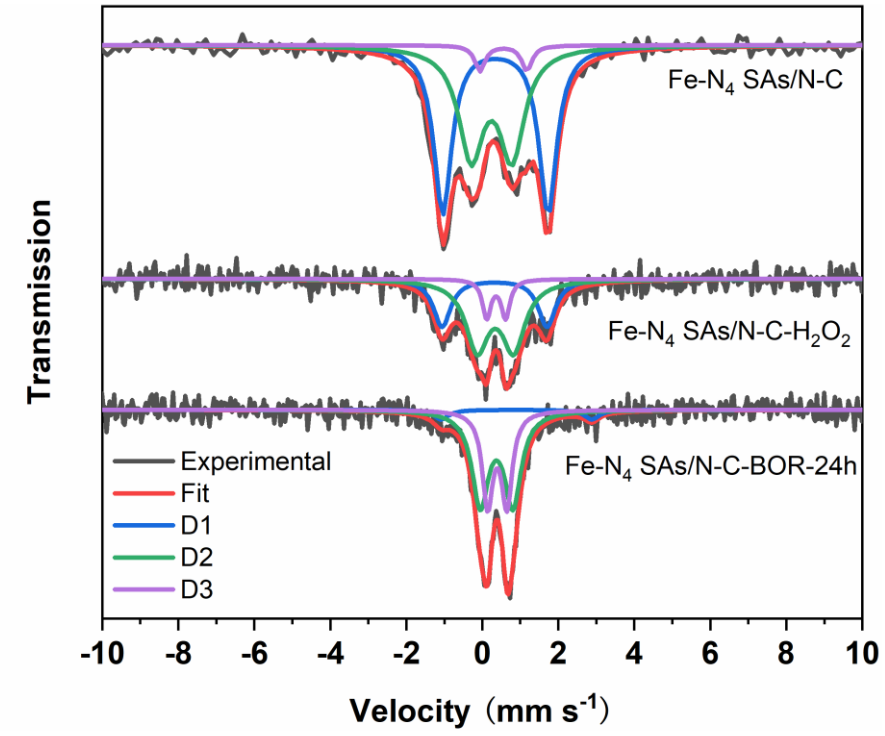


**Supplementary Figure 25. $^{57}$Fe Mössbauer spectra characterizations.** $^{57}$Fe Mössbauer spectra of the Fe-$N_4$ SAs/N-C catalyst after $H_2O_2$ treatment for 12 h and after BOR for 24 h in comparison to the original sample.

## Supplementary Tables

**Supplementary Table 1.** The elemental compositions of Fe-$N_4$ SAs/N-C, Fe-$N_3C_1$ SAs/N-C, and Fe-$N_2C_2$ SAs/N-C catalysts estimated from ICP and element analysis*.

| Catalyst | Fe (wt. %) | C (wt. %) | N (wt. %) | H (wt. %) | O (wt. %) |
|---|---|---|---|---|---|
| Fe-$N_4$ SAs/N-C | 3.18 | 50.69 | 23.48 | 2.10 | 20.55 |
| Fe-$N_3C_1$ SAs/N-C | 3.46 | 54.42 | 19.35 | 1.71 | 21.06 |
| Fe-$N_2C_2$ SAs/N-C | 3.06 | 60.79 | 17.87 | 1.01 | 17.27 |
| Fe-$N_2C_2$ SAs/N-C-$NH_3$ | 3.12 | 52.42 | 21.35 | 1.86 | 21.25 |

*C, N and H contents were detected by element analysis, Fe contents were determined by ICP, and O contents were obtained by subtraction method.

**Supplementary Table 2.** Structural parameters of the Fe-$N_xC_y$ SAs/N-C catalysts extracted from the EXAFS fitting. ($S0^2$=0.80).

| | Scattering pair | CN | R(Å) | $\sigma^2(10^{-3}Å^2)$ | $\Delta E_0$(eV) | R factor |
|---|---|---|---|---|---|---|
| Fe-$N_4$ SAs/N-C | Fe -N | 3.9±0.8 | 1.93±0.02 | 6.9±0.9 | 5.0±1.0 | 0.0054 |
| Fe-$N_3C_1$ SAs/N-C | Fe -N | 3.3±0.7 | 1.93±0.02 | 7.2±1.1 | 4.5±1.7 | 0.0069 |
| | Fe -C | 0.8±0.6 | 1.92±0.02 | 5.8±0.9 | 6.1±1.5 | 0.0059 |
| Fe-$N_2C_2$ SAs/N-C | Fe -N | 2.2±0.8 | 1.92±0.02 | 6.9±3.9 | 3.6±1.5 | 0.0051 |
| | Fe -C | 1.9±0.5 | 1.92±0.02 | 5.9±3.6 | 5.4±1.3 | 0.0062 |
| Fe-$N_2C_2$ SAs/N-C-$NH_3$ | Fe-N | 3.5±0.8 | 1.93±0.02 | 7.9±1.4 | 3.9±0.8 | 0.0068 |

$S0^2$ is the amplitude reduction factor; CN is the coordination number; R is interatomic distance (the bond length between central atoms and surrounding coordination atoms); $\sigma^2$ is Debye-Waller factor (a measure of thermal and static disorder in absorber-scatterer distances); $\Delta E_0$ is edge-energy shift (the difference between the zero kinetic energy value of the sample and that of the theoretical model). R factor is used to value the goodness of the fitting.

**Supplementary Table 3.** Comparison of BOR performance of the Fe-$N_4$ SAs/N-C catalyst with various reported catalysts.

| Catalyst | Oxidant | Temp. (°C) | Time (h) | Benzene Conv. (%) | Phenol Yield (%) | Phenol Sel. (%) | Ref. |
|---|---|---|---|---|---|---|---|
| Fe-$N_4$ SAs/N-C | $H_2O_2$ | 30 | 24 | 78.4 | 78.4 | 100 | This work |
| Fe-$N_3C_1$ SAs/N-C | $H_2O_2$ | 30 | 24 | 72.2 | 71.1 | 98.5 | This work |
| Fe-$N_2C_2$ SAs/N-C | $H_2O_2$ | 30 | 24 | 16.2 | 15.4 | 94.8 | This work |
| Fe-$N_2C_2$ SAs/N-C-$NH_3$ | $H_2O_2$ | 30 | 24 | 73.6 | 73.6 | 100 | This work |
| Fe(II) Complex | $H_2O_2$ | 50 | 3 | 65 | 64 | 98.5 | 1 |
| SA-Fe/CN | $H_2O_2$ | 60 | 24 | 45 | 42.3 | 94 | 2 |
| VPO@GO | $H_2O_2$ | 60 | 8 | 32.8 | 32.8 | 100 | 3 |
| $H_4PMo_{11}VO_{40}$ | $H_2O_2$ | RT | 1.7 | 26 | 23.6 | 90.7 | 4 |
| Ni(II) Complex | $H_2O_2$ | 60 | 5 | 23 | 21 | 91.3 | 5 |
| $FeN_4$/GN | $H_2O_2$ | RT | 24 | 23.4 | 18.7 | 79.9 | 6 |
| graphene | $H_2O_2$ | 60 | 16 | 18 | 17.8 | 99 | 7 |
| 4Cu/MCM-41 | $H_2O_2$ | RT | 1.67 | 21 | 19.7 | 94 | 8 |
| Titanium Silicalite | $H_2O_2$ | 100 | 2 | 8.6 | 8.1 | 94 | 9 |
| Fe-g-$C_3N_4$/SBA-15 | $H_2O_2$ | Light | 4 | 11.9 | 2.5 | 16.8 | 10 |
| h-BCN nanosheets | $H_2O_2$ | Light | 2 | 15.9 | 14 | 88 | 11 |
| CNT7000 | $H_2O_2$ | 60 | 6 | 6.4 | 5.8 | 91.5 | 12 |

**Supplementary Table 4.** Relative energy (in eV) with ZPE included along the reaction profile.

| | IS | MS1 | MS2 | MS3 | TS1 | MS4 | MS5 | MS6 | TS2 | MS7 | TS3 | MS8 | FS |
|---|---|---|---|---|---|---|---|---|---|---|---|---|---|
| $Fe\text{-}N_4$ | 0.00 | -2.14 | -1.68 | -2.28 | -2.17 | -3.48 | -2.98 | -4.00 | -2.94 | -3.67 | -3.10 | -5.66 | -4.45 |
| $Fe\text{-}N_3C_1$ | 0.00 | -2.47 | -2.00 | -2.41 | -2.27 | -3.57 | -3.04 | -4.47 | -2.94 | -3.71 | -3.19 | -5.89 | -4.77 |
| $Fe\text{-}N_2C_2$ | 0.00 | -3.01 | -2.56 | -2.93 | -2.56 | -3.34 | -2.78 (-5.14)[a] | -3.83 | -2.79 | -4.06 | -3.70 | -6.47 | -5.33 |

[a] the relative total energy of MS5′ with reference to $Fe\text{-}N_2C_2$ SAs/N-C catalyst.

**Supplementary Table 5.** Energy barriers of each transition steps in terms of the quantum-mechanical total energy and Gibbs free energy at T= 298.15 K.

| | Total energy barrier (ZPE included) /eV | | | Free energy barrier (ZPE included)/eV | | |
|---|---|---|---|---|---|---|
| | $Fe\text{-}N_4$ | $Fe\text{-}N_3C_1$ | $Fe\text{-}N_2C_2$ | $Fe\text{-}N_4$ | $Fe\text{-}N_3C_1$ | $Fe\text{-}N_2C_2$ |
| TS1 | 0.71 | 0.14 | 0.37 | 0.07 | 0.11 | 0.30 |
| TS2 | 1.06 | 1.53 | 1.04 | 1.13 | 1.49 | -0.06 |
| TS3 | 0.57 | 0.52 | 0.36 | 0.56 | 0.44 | 0.30 |
| TS2′ | 1.34 | 1.12 | -- | 1.47 | 1.23 | -- |
| TS3′ | -- | -- | 2.17 | -- | -- | 2.16 |

**Supplementary Table 6.** Summary of the Mössbauer parameters and assignments to different iron species in Fe-$N_4$ SAs/N-C, Fe-$N_4$ SAs/N-C-$H_2O_2$ and Fe-$N_4$ SAs/N-C-BOR-24h catalysts.

| Catalyst | Component | IS / mm $s^{-1}$ | QS / mm $s^{-1}$ | LW / mm $s^{-1}$ | area %[a] | Assignment[b] |
|---|---|---|---|---|---|---|
| Fe-$N_4$ SAs/N-C | D1 | 0.344 | 2.773 | 0.572 | 50.46 | $Fe^{II}N_4$, MS |
| | D2 | 0.243 | 1.079 | 0.794 | 45.07 | N-($Fe^{III}N_4$)-N, LS |
| | D3 | 0.557 | 1.233 | 0.331 | 4.44 | X-$Fe^{III}N_4$-Y, (X, Y ligands like O/N), HS |
| Fe-$N_4$ SAs/N-C-$H_2O_2$ | D1 | 0.320 | 2.762 | 0.557 | 30.669 | $Fe^{II}N_4$, MS |
| | D2 | 0.344 | 0.960 | 0.734 | 57.349 | N-($Fe^{III}N_4$)-N, LS |
| | D3 | 0.365 | 0.497 | 0.270 | 11.981 | X-$Fe^{III}N_4$-Y, HS |
| Fe-$N_4$ SAs/N-C-BOR-24h | D1 | 0.900 | 3.969 | 0.485 | 5.502 | $Fe^{II}N_4$, MS |
| | D2 | 0.375 | 0.860 | 0.529 | 57.258 | N-($Fe^{III}N_4$)-N, LS |
| | D3 | 0.394 | 0.507 | 0.344 | 37.238 | X-$Fe^{III}N_4$-Y, HS |

[a]The relative absorption area of each iron species in Fe-N-C samples.

[b]LS, MS, and HS denote low-spin, medium-spin, and high-spin, respectively.

**Supplementary References**


1. Carneiro, L., Silva, A. R. Selective direct hydroxylation of benzene to phenol with hydrogen peroxide by iron and vanadyl based homogeneous and heterogeneous catalysts. *Catal. Sci. Technol.* **6**, 8166-8176 (2016).

2. Zhang, M. et al. Metal (hydr) oxides@ polymer core-shell strategy to metal single-atom materials. *J. Am. Chem. Soc.* **139**, 10976-10979 (2017).

3. Borah, P., Datta, A., Nguyen, K. T., Zhao, Y. $VOPO_4 \cdot 2H_2O$ encapsulated in graphene oxide as a heterogeneous catalyst for selective hydroxylation of benzene to phenol. *Green Chem.* **18**, 397-401 (2016).

4. Zhang, J., Tang, Y., Li, G., Hu, C. Room temperature direct oxidation of benzene to phenol using hydrogen peroxide in the presence of vanadium-substituted heteropolymolybdates. *Appl. Catal. A-Gen.* **278**, 251-261 (2005).

5. Morimoto, Y., Bunno, S., Fujieda, N., Sugimoto, H., Itoh, S. Direct hydroxylation of benzene to phenol using hydrogen peroxide catalyzed by nickel complexes supported by pyridylalkylamine ligands. *J. Am. Chem. Soc.* **137**, 5867-5870 (2015).

6. Deng, D. et al. A single iron site confined in a graphene matrix for the catalytic oxidation of benzene at room temperature. *Sci. Adv.* **1**, e1500462 (2015).

7. Yang, J. H. et al. Direct catalytic oxidation of benzene to phenol over metal-free graphene-based catalyst. *Energy Environ. Sci.* **6**, 793-798 (2013).

8. Parida, K. M., Rath, D. Structural properties and catalytic oxidation of benzene to phenol over CuO-impregnated mesoporous silica, *Appl. Catal. A-Gen.* **321**, 101-108 (2007).

9. Balducci, L. et al. Direct oxidation of benzene to phenol with hydrogen peroxide over a modified titanium silicalite, *Angew. Chem.* **115**, 5087-5090 (2003).

10. Chen, X., Zhang, J., Fu, X., Antonietti, M., Wang, X. Fe-g$C_3N_4$-catalyzed oxidation of benzene to phenol using hydrogen peroxide and visible light. *J. Am. Chem. Soc.* **131**, 11658-11659 (2009).

11. Wang, B. et al. Direct hydroxylation of benzene to phenol on h-BCN nanosheets in the presence of $FeCl_3$ and $H_2O_2$ under visible light, *Catal. Today,* 324, 73-82 (2018).

12. Wen, G., Wu, S., Li, B., Dai, C., Su, D. S. Active sites and mechanisms for direct oxidation of benzene to phenol over carbon catalysts. *Angew. Chem. Int. Ed.* **54**, 4105-4109 (2015).
